# The Efficient and Environmentally Friendly Chlorination of Arene, Alcohol, Halobenzene, and Peroxide Catalyzed by Fe–Ba Binary Oxides Using Hydrochloric Acid as Chlorine Source and Aqueous H_2_O_2_ as Oxidant

**DOI:** 10.3390/molecules29225451

**Published:** 2024-11-19

**Authors:** Sidra Chaudhary, Qin Pan, Yong Wu, Zainab Bibi, Xiaoyong Li, Qinxiang Jia, Yang Sun

**Affiliations:** 1 Department of Applied Chemistry, School of Chemistry, Xi’an Jiaotong University, No. 28, Xianning West Road, Xi’an 710049, China; sidra-ch576@stu.xjtu.edu.cn (S.C.); panqin202212@163.com (Q.P.); specwy@mail.xjtu.edu.cn (Y.W.); zainabbibi55@stu.xjtu.edu.cn (Z.B.); lixy6658@xjtu.edu.cn (X.L.); qinxiangjia1984@mail.xjtu.edu.cn (Q.J.); 2 Xi’an Biomass Green Catalysis and Advanced Valorization International Science and Technology Cooperation Base, No. 28, Xianning West Road, Xi’an 710049, China

**Keywords:** chlorination, Fe–Ba binary oxide, hydrogen peroxide, oxidant, conversion

## Abstract

A series of Fe–Ba mixed oxides, including a pure Fe-containing sample as a reference, have been synthesized via a sol–gel process using Fe^3+^ or Fe^2+^ salts and BaSO_4_ as raw materials, with Pluronic P123 serving as a template. These oxides have been thoroughly characterized and subsequently utilized as catalysts for the chlorination of various organic molecules. Commercial hydrochloric acid, known for its relative safety, and environmentally friendly aqueous hydrogen peroxide were employed as the chlorine source and oxidant, respectively. The pure Fe-containing catalyst displays excellent thermal stability between 600 and 800 °C and exhibited moderate to high conversions in the chlorination of toluene, benzene, and *tert*-butyl hydroperoxide, with remarkable *ortho*-selectivity in chlorination of toluene. The combination of Fe^3+^ salt with BaSO_4_ in the sol–gel process results in a Fe–Ba mixed oxide catalyst composed of BaO_2_, BaFe_4_O_7_, and Fe_2_O_3_, significantly enhancing the chlorination activity compared to that displayed by the pure Fe catalyst. Notably, the chlorination of *tert*-butyl hydroperoxide (TBHP) does not require additional oxidants such as H_2_O_2_, and involves both electrophilic substitution and nucleophilic addition. Notably, the chlorination of bromobenzene yields chlorobenzene as the sole product, a transformation that has not been previously reported. Overall, this catalytic chlorination system holds promise for advancing the chlorination industry and enhancing pharmaceutical production.

## 1. Introduction

Chlorine-containing compounds have emerged as highly functional materials in pharmaceutical and other industrial fields, garnering extensive and sustained interest over the years [1]. In pharmaceuticals, the incorporation of chlorine atoms into specific positions of biologically active molecules has been shown to significantly enhance their intrinsic biological activity. This enhancement occurs because the electrophilic reactivity of the carbon center adjacent to the chlorine atom is improved, facilitating the replacement of chlorine by nucleophiles, which ultimately influences the biological properties of the pharmaceuticals [2]. Additionally, the introduction of chlorine into a bioactive molecule increases its lipophilicity, thereby enhancing its ability to traverse cell membranes and leading to improved therapeutic effects at lower dosages. Finally, it is important to note that organochlorines are not inherently toxic or harmful to the environment; their safety largely depends on dosage and application scenarios [3].

**Figure 1:**
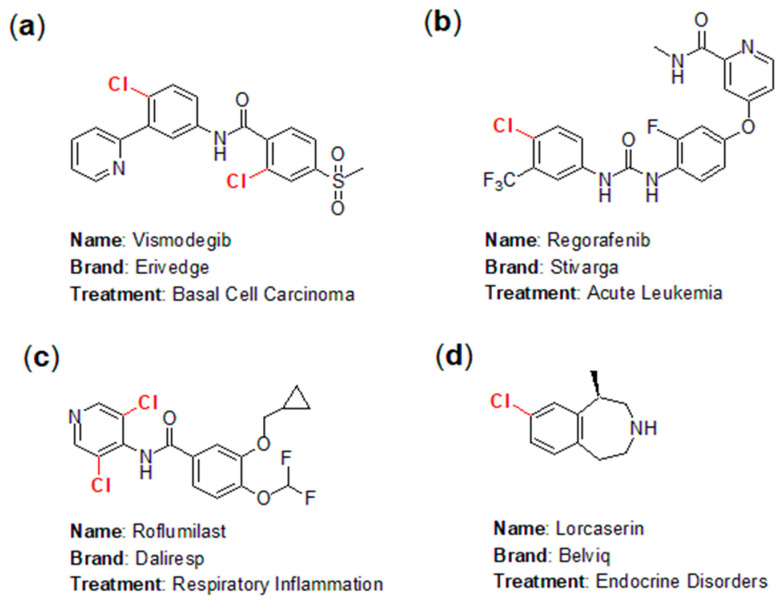
Representative chlorine-containing pharmaceuticals: (**a**) Vismodegib; (**b**) Regorafenib; (**c**) Roflumilast; (**d**) Lorcaserin.

The application of chlorine in the pharmaceutical industry has emerged as one of the fastest-growing areas in chemistry, highlighting the fascinating and instructive role of halogens in pharmaceutical development. In practice, numerous chlorine-containing pharmaceuticals have been approved for the market, yielding significant economic benefits. Notable examples include Vismodegib (marketed as Erivedge by Genentech; Figure 1a), an effective inhibitor of the hedgehog signaling pathway for the treatment of basal cell carcinoma (BCC) [4]; Regorafenib (branded as Stivarga; Figure 1b), used for treating acute leukemia [5]; Roflumilast (sold as Daliresp; Figure 1c), a selective phosphodiesterase type 4 inhibitor aimed at reducing respiratory inflammation [6]; and Lorcaserin (known as Belviq; Figure 1d), a serotonin 5-HT2C receptor agonist used to treat endocrine disorders [7].

At the same time, chlorinated compounds are ubiquitous in industrialized societies found in materials such as plastics [8], polymers [9], and solvents [10]. Chlorination significantly modifies and enhances the physical and chemical properties of hydrocarbons. In general, chlorine-containing compounds play a crucial role in modern medicine and industry, and they are likely to remain irreplaceable for the foreseeable future [11].

On the other hand, the development of new approaches for developing functional and useful molecules has garnered significant and sustained attention in both academic and industrial communities for at least the past thirty years. Catalytic C–H bond functionalization has emerged as an efficient and powerful method for constructing sophisticated molecules, surpassing traditional synthetic methods. This technique avoids the challenges associated with sourcing specific raw materials, managing complex reaction conditions, and enduring lengthy reaction processes [12]. Furthermore, catalytic C–H bond functionalization utilizes simpler hydrocarbons or polymers, as well as more readily available synthons with active groups, as starting materials [12]. This approach enables synthetic reactions to be completed in a shorter timeframe, significantly reducing production costs. There remains considerable potential for further developing new methods for catalytic C–H bond functionalization.

Among the various approaches to C–H bond activation, the formation of C–Cl bonds is crucial for synthesizing a wide range of chlorine-containing pharmaceuticals and other industrial products. Several chlorine sources have been developed for the chlorination of organic molecules. Initially, molecular halogens such as Cl_2_ emerged as efficient chlorinating agents, primarily sourced from the chloro–alkali industry [13]. However, Cl_2_ is highly toxic and can be explosively reactive, posing significant operational risks [14]. Additionally, its use is often impractical in regions lacking chloro–alkali facilities.

To address the limitations of Cl_2_, phosphorus chlorides such as POCl_3_ [15] and PCl_5_ [16] have been introduced for chlorination. However, these compounds are acidic, corrosive, and sensitive to moisture, which not only makes their handling hazardous but also leads to the formation of undesirable by-products. These by-products primarily arise from side reactions or dehydration involving hydroxyl groups in the substrate [17].

Furthermore, the combined use of hydrogen chloride (HCl) and hydrogen peroxide (H_2_O_2_) has emerged as a more sustainable halogenating agent, inspired by the activity of haloperoxidase enzymes that catalyze halogenation reactions through the oxidation of halides with H_2_O_2_ [18]. However, this approach faces two significant challenges. First, a large dosage of HCl is required to ensure complete conversion of the substrate. Second, the substrate scope is limited to only a few reactive compounds [18]. Currently, efforts to optimize this method appear to be focused on the use of specific solvents, such as polyfluorinated alcohols, which can enhance the activation of H_2_O_2_ [18].

**Figure 2:**
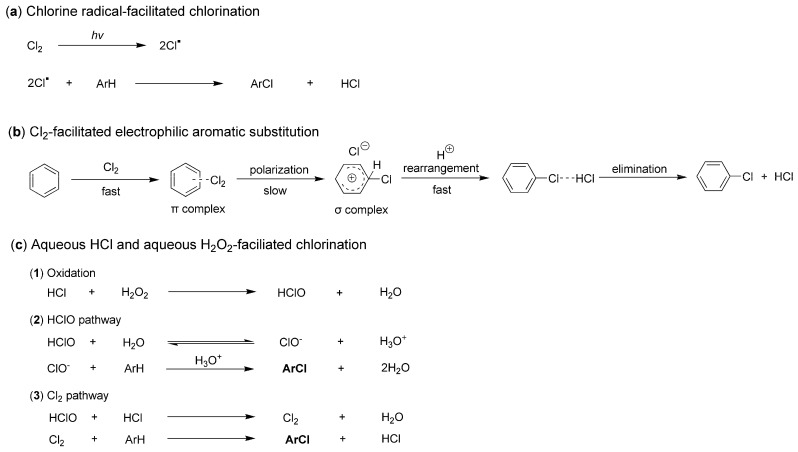
Three known chlorination processes facilitated by different chlorine sources.

It is also intriguing to explore the chlorination processes facilitated by different chlorine sources. However, significant controversies remain regarding the precise details of this transformation. When Cl_2_ is employed as a chlorine source, it is believed that chlorine radicals (Cl·) can be generated through photoexcitation. These radicals then react with organic molecules, such as arenes, resulting in the formation of chlorinated compounds (Figure 2a) [19]. Additionally, there exists an electrophilic aromatic substitution (EAS) pathway that describes the chlorination of arenes without the involvement of chlorine radicals [20]. For instance, Cl_2_ can rapidly react with benzene to form a π-complex, which subsequently undergoes a slow rearrangement to yield a σ-complex. This σ-complex then rearranges further, leading to the loss of HCl and the formation of the final product, chlorobenzene (Figure 2b) [20].

When chlorination is facilitated using hydrogen chloride (HCl) as the chlorine source, the underlying process primarily involves the effects of haloperoxidase enzymes, typically stimulated by H_2_O_2_. Initially, HCl is oxidized by H_2_O_2_ to form hypochlorous acid (HClO), which subsequently reacts with additional HCl to produce active Cl_2_. These oxidized species, including HClO and Cl_2_, can then react in situ with substrates such as arenes to yield chlorinated products (Figure 2c) [18].

On the other hand, the use of catalysts plays a crucial role in the chlorination of organic molecules, addressing several important aspects of this transformation. These include safer chlorine sources with reduced dosages, enhanced conversion efficiency, a broader substrate scope, improved stereoselectivity, and the development of recyclable and eco-friendly catalysts.

In this context, palladium catalysts and their applications in chlorination have attracted significant and ongoing attention. For instance, the PdCl_2_-catalyzed chlorination of *N*-quinolinylbenzamide derivatives demonstrates high *ortho*-selectivity when HCl is used as the chlorine source under anodic oxidation conditions, converting Cl^−^ into ClO^−^ through an intermediate with Pd^2+^-coordinated substrate [21].

Furthermore, PdCl_2_(MeCN)_2_ exhibits high regioselectivity in the synthesis of dichlorinated tetrahydroquinolines using CuCl_2_ as the chlorine source, facilitated by a chloropalladation-initiated cascade process [22]. Additionally, Brønsted acids such as trifluoromethanesulfonic acid (HOTf) and trifluoroacetic acid (TFA) have proven effective as ligands for stabilizing Pd(OAc)_2_ in the catalytic chlorination of phenol carbamates when *N*-chlorosuccinimide (NCS) is employed as the chlorine source [23]. These findings indicate that palladium salts not only coordinate with substrates but also oxidize Cl^−^ into ClO^−^ (or Cl_2_) in situ, thereby enhancing the chlorination of organic molecules.

Iron(III) triflimide (Fe(NTf_2_)_3_) has been synthesized by combining FeCl_3_ with [Bmim][NTf_2_]. This compound was subsequently utilized as a catalyst, with *N*-chlorosuccinimide (NCS) serving as the chlorine source, for the chlorination of a diverse array of anisole, aniline, acetanilide, and phenol derivatives. This approach resulted in high regioselectivity and yields [24]. Traditionally, since the chlorination of aromatic compounds involves electrophilic aromatic substitution [20], it can be inferred that Fe^3+^ may also oxidize Cl^−^ (or the chloro group) into ClO^−^ (or Cl_2_) in situ, similar to the behavior of Pd^2+^.

Additionally, other various metal and non-metal catalysts, including rhodium [25], selenide [26], and alkali chloride [27] catalysts, have been proposed for chlorination reactions. These catalysts generally demonstrate high chemoselectivity and stereoselectivity, along with notable substrate specificity. In light of environmental protection concerns, several organic catalysts have also been explored for facilitating chlorination. These include sulfoxide [28], chloramine-T [29], and *p*-toluenesulfonic acid (*p*-TsOH) [30]. When combined with various chlorine sources, these organic catalysts provide satisfactory results in chlorination reactions.

While various chlorination catalysts have been developed that achieve high transformation efficiencies and excellent stereoselectivities, several significant drawbacks remain for their large-scale application and the production of chlorinated compounds.

Firstly, many of the known metal and non-metal catalysts are prohibitively expensive and non-recyclable, which significantly increases production costs. Secondly, the majority of these catalysts are homogeneous, making it difficult to remove residues and posing a risk of contaminating the chlorinated products. Additionally, many existing chlorination catalysts require costly, toxic, and structurally complex chlorine sources. This not only raises production costs but also generates unwanted by-products and complicates waste disposal. Lastly, most of the known catalysts are effective only for a limited range of specific substrates, indicating a need to broaden their substrate scope for more versatile applications. Therefore, there is significant potential for exploring new chlorination catalysts to facilitate the large-scale production of chlorinated products.

From another perspective, it is noteworthy and instructive that sophisticated and expensive complex catalysts can be replaced by more synthetically convenient and functionalized oxides containing the same metal or active components. This approach not only reduces catalyst production costs but also creates heterogeneous catalysts, which facilitate product purification. In this context, the original function of the ligands used in complex catalysts can be compensated for by the additives incorporated during the synthesis of oxide catalysts. Moreover, metal oxides are typically more insoluble in both water and organic solvents than metal complexes, which significantly enhances the heterogeneity of oxide chlorination catalysts.

Furthermore, the choice of catalyst support materials deserves attention. Firstly, these materials can enhance the reactivity of the catalytically active components. Secondly, incorporating support materials may further increase the overall heterogeneity of the catalyst. For example, in addition to well-known supports such as SiO_2_ and Al_2_O_3_ [31], barium sulfate (BaSO_4_) appears to be a promising candidate for supporting catalytically active components. BaSO_4_ is typically white or colorless, heat-resistant (with a melting point of 1580 °C), chemically inert (insoluble in concentrated acids or alkalis), and almost insoluble in water (1.10 × 10^−10^ mol L^−1^, 25 °C), with a high density of 4.50 g cm^−3^ [32]. These properties enable BaSO_4_ to withstand harsh chemical environments and protect active components during catalysis, while its high density facilitates convenient catalyst precipitation.

Secondly, although BaSO_4_ contains “barium” which is often associated with “heavy” metals, this substance is not classified as a “toxic” compound, primarily due to its low solubility [33]. Lastly, the porous nature of BaSO_4_ crystals allows them to adsorb foreign ions and demonstrates a tendency for co-precipitation [32]. Therefore, the immobilization of active components on BaSO_4_ crystals appears to be both reasonable and plausible during the synthesis of heterogeneous catalysts.

The establishment of a more efficient and environmentally friendly system for the chlorination of organic molecules with a broader substrate scope has been attempted. To achieve this, a pure iron oxide and three Fe–Ba mixed oxides using Pluronic P123 as both a pore-forming and functionalizing agent have been prepared through a sol–gel process. Following comprehensive characterization, these oxides were utilized as catalysts in the catalytic chlorination of arenes, alcohols, halobenzenes, and peroxides, with commercial hydrochloric acid serving as the chlorine source and aqueous H_2_O_2_ as the oxidant.

High accessibility of catalysts was achieved, resulting in chlorinated products that are both highly useful and valuable chemical intermediates. Additionally, hydrochloric acid and aqueous H_2_O_2_ were utilized as significantly safer and more cost-effective raw materials for chlorination compared to traditional reagents such as Cl_2_, POCl_3_, or *N*-chlorosuccinimide (NCS). Consequently, a meaningful advancement in the chlorination industry and the associated pharmaceutical manufacturing processes has been demonstrated.

## 2. Results and Discussion

### 2.1. Synthesis of Catalysts

**Figure 3:**
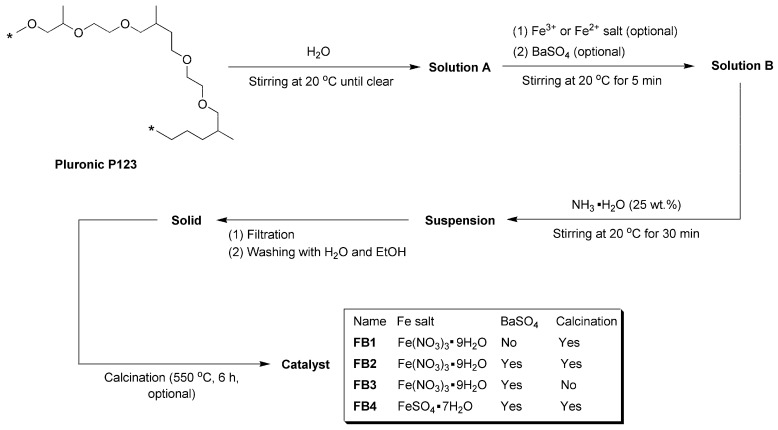
Synthesis of catalysts FB1–FB4 (asterisks in structure of Pluronic P123 means repetitive units).

As illustrated in Figure 3, Pluronic P123 has been utilized as a structure-directing agent in the synthesis of SBA-15, demonstrating excellent pore-forming properties [34]. Consequently, this compound was dissolved in water to create Solution A, which served as a template for the synthesis of oxide catalysts. Following the addition of Fe^3+^ or Fe^2+^ salts and commercial BaSO_4_ particles, the resulting mixture, referred to as Solution B, was treated with NH_3_·H_2_O (25 wt.%), generating an alkaline environment. This facilitated the rapid hydrolysis of Fe^3+^ or Fe^2+^ into Fe(OH)_3_ or Fe(OH)_2_, which subsequently precipitated onto BaSO_4_. The suspension was then filtered under reduced pressure, and the resulting solids were meticulously washed with distilled water and ethanol to eliminate any adsorbed Pluronic P123. After calcination at 550 °C for 6 h, aimed at complete dehydration of Fe(OH)_3_ or Fe(OH)_2_ into oxides and removing all organic species, including Pluronic P123, while preserving the pores, the catalysts (FB1–FB4, Figure 3) were obtained in powdered form.

### 2.2. Characterization of Synthesized Catalysts

#### 2.2.1. Elemental and Crystalline Analysis

**Figure 4:**
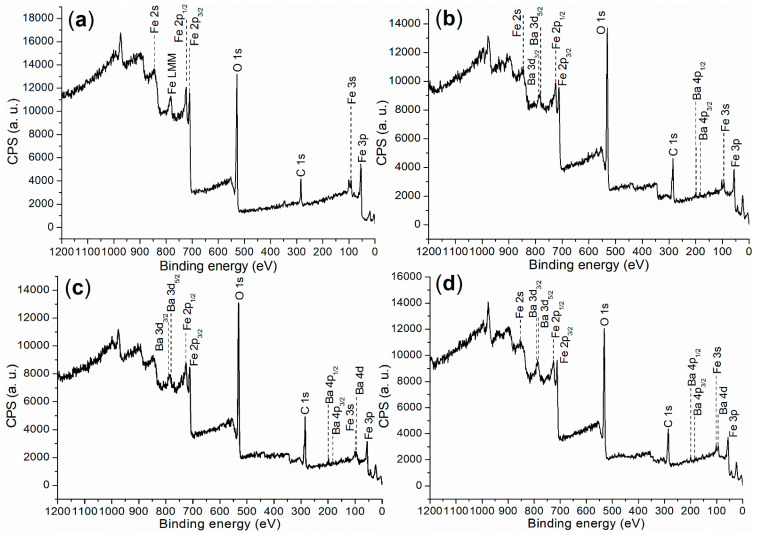
XPS survey scan for the synthesized catalysts: (**a**) FB1; (**b**) FB2; (**c**) FB3; (**d**) FB4.

X-ray photoelectron spectroscopy (XPS) was initially utilized to investigate the elemental composition and chemical states on the surfaces of the synthesized catalysts, probing a depth of 0–3 nm. The XPS survey scan is presented in Figure 4, with the corresponding binding energies and atomic compositions detailed in Table 1.

First, FB1 exhibited significantly higher levels of Fe and O, while showing no detectable Ba content compared to FB2–FB4 (Table 1). This suggests that both Fe^3+^ and Fe^2+^ ions were incorporated into the BaSO_4_ framework during the formation of FB2–FB4 (Figure 3). Additionally, the carbon content on the surface of FB1 was notably lower than that on the surfaces of FB2–FB4 (Table 1), which likely indicates that BaSO_4_ retained more carbon species derived from Pluronic P123 during calcination at 550 °C (Figure 3).

Next, FB2 exhibited a higher oxygen content, along with lower levels of Fe and Ba, and a slightly increased carbon content compared to FB3 (Table 1). This suggests that the calcination process at 550 °C facilitated the oxidation of metal- or carbon-containing species, resulting in the incorporation of more oxygen into the synthesized catalyst (Figure 3).

Furthermore, the surface contents of Fe and Ba in FB4 are significantly higher than those in FB2 (Table 1). This likely indicates that using Fe^2+^ salt as a raw material enhances the hydrolysis of Fe^2+^, leading to the formation of Fe(OH)_2_ or Fe(OH)_3_ more effectively than using Fe^3+^ salt as a raw material.

**Figure 5:**
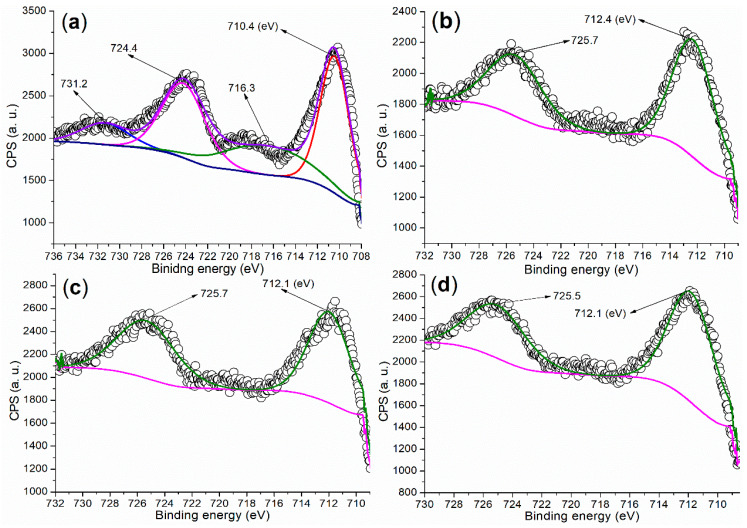
XPS measurements of the Fe 2p regions for synthesized catalysts: (**a**) FB1; (**b**) FB2; (**c**) FB3; (**d**) FB4.

It is crucial to investigate the chemical state of metal elements on the catalyst surface to better understand the active components during catalysis. The Fe 2p regions of the synthesized catalysts are presented in Figure 5. Firstly, the Fe 2p region of FB1 (Figure 5a) displays two doublets consisting of four peaks. The peaks at 724.4 eV and 710.4 eV (the first doublet) correspond to Fe 2p_1/2_ and 2p_3/2_ photoelectrons, respectively, which are attributed to Fe^3+^ ions fixed within the Fe_2_O_3_ lattice [35]. The second doublet, appearing at 731.2 eV and 716.3 eV, represents satellite peaks, further confirming the presence of Fe 2p_1/2_ and 2p_3/2_ photoelectrons and indicating the formation of the Fe_2_O_3_ phase [35].

In contrast, if the dominant product were Fe_3_O_4_, we would expect to see no satellite peaks [35]. Notably, the peak centered at 710.4 eV extends into the region with binding energies lower than 710 eV (Figure 5a). This extension may characterize Fe(II)−O and Fe(III)−O bonds in Fe_3_O_4_ (magnetite) [35], suggesting that FB1 may still contain a small amount of Fe_3_O_4_.

**Figure 6:**
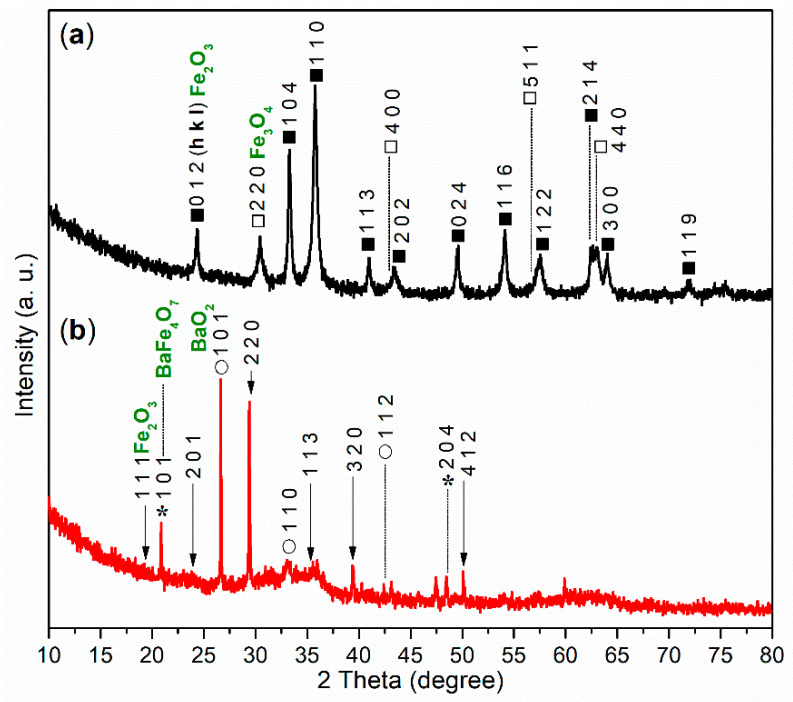
Wide-angle XRD diffractograms (2*θ* = 10°–80°): (**a**) FB1 (dark cubes, Fe_2_O_3_; white cubes, Fe_3_O_4_); (**b**) FB2 (arrows, Fe_2_O_3_; asterisks, BaFe_4_O_7_; circles, BaO_2_).

The XRD experiment was conducted to further analyze the composition of FB1. As illustrated in Figure 6a, the wide-angle XRD pattern (2*θ* = 10°–80°) of FB1 reveals two distinct diffraction systems. The first system corresponds to Fe_2_O_3_ (dark cubes, hematite, *α*-Fe_2_O_3_, PDF no. 33-0664), while the second system indicates the presence of Fe_3_O_4_ (white cubes, magnetite, PDF no. 19-0629). This result clearly aligns with the findings from the XPS studies (Figure 5a).

On the other hand, FB2 displays two peaks at 725.7 eV and 712.4 eV in the Fe 2p region, corresponding to Fe 2p_1/2_ and 2p_3/2_ photoelectrons, respectively, without any significant satellite peaks (Figure 5b). These peak values are notably higher than those observed in FB1 (Figure 5b vs. Figure 5a). Additionally, the peak centered at 712.4 eV (red shadow) in the Fe 2p region of FB2 does not extend into the area with binding energies below 710 eV. This suggests that FB2 does not contain *α*-Fe_2_O_3_, unlike FB1, nor does it contain Fe_3_O_4_ [35].

The XRD analysis further supports this conclusion. As shown in Figure 6b, there are no diffraction peaks corresponding to *α*-Fe_2_O_3_ or Fe_3_O_4_. Instead, peaks for Fe_2_O_3_ (arrows, iron oxide, PDF no. 65-0390), BaFe_4_O_7_ (asterisks, barium iron oxide, PDF no. 25-1476), and BaO_2_ (circles, barium oxide, PDF no. 07-0233) are present (Figure 6b).

Furthermore, FB3 and FB4 exhibit very similar Fe 2p regions, with peaks corresponding to Fe 2p_1/2_ photoelectrons appearing at 725.7–725.5 eV, while those for Fe 2p_3/2_ photoelectrons are both observed at 712.1 eV (Figure 5c,d). Notably, no satellite peaks are present in their Fe 2p regions. Additionally, the peak for Fe 2p_3/2_ photoelectrons in both FB3 and FB4 extends into the area with binding energies below 710 eV (red shadows, Figure 5c,d). Therefore, it is likely that FB3 and FB4 contain Fe_3_O_4_ [35].

**Figure 7:**
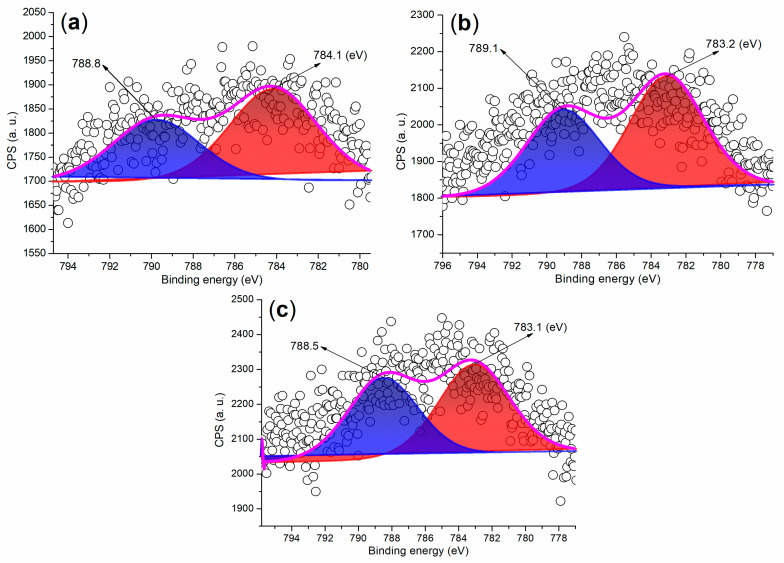
XPS measurements of the Ba 3d regions for synthesized catalysts: (**a**) FB2; (**b**) FB3; (**c**) FB4.

The Ba 3d regions of the synthesized catalysts are depicted in Figure 7. As illustrated in Figure 7a, FB2 displays two partially overlapping peaks at 788.8 eV and 784.1 eV, which correspond to the Ba 3d_3/2_ and 3d_5/2_ photoelectrons, respectively. These peaks primarily arise from Ba^2+^ ions fixed in a mixed oxide phase [36]. In contrast, previous studies have reported that BaSO_4_ nanoparticles exhibit two distinct and sharp peaks at 794.78 eV (Ba 3d_3/2_) and 779.48 eV (Ba 3d_5/2_) in the Ba 3d region [37]. Moreover, FB3 and FB4 reveal Ba 3d peaks that closely resemble those of FB2 (Figure 7b,c compared to Figure 7a). This indicates that the BaSO_4_ particles utilized as raw materials in this study likely underwent a transformation into a mixed oxide phase under alkaline conditions, either through the hydrolysis of Ba^2+^ ions or via calcination during the catalyst synthesis process (refer to Figure 3).

**Figure 8:**
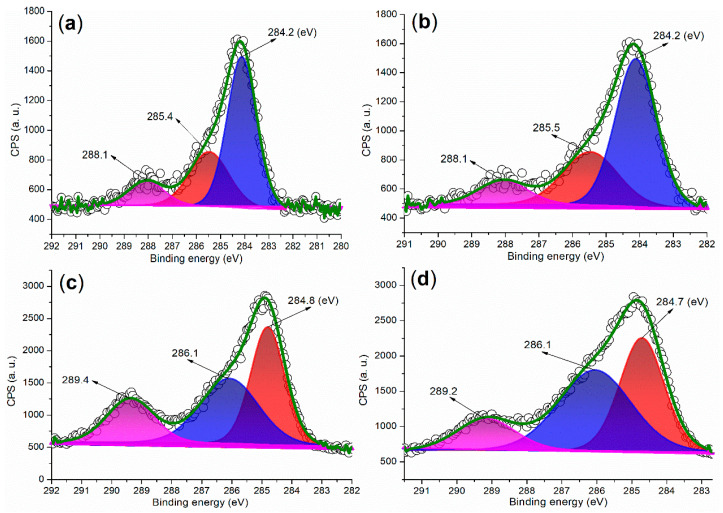
XPS measurements of the C 1s region: (**a**) FB1; (**b**) FB2; (**c**) FB3; (**d**) FB4.

Figure 8 presents the C 1s regions of the synthesized catalysts. In the case of FB1 (Figure 8a), three peaks are observed at 284.2 eV, 285.4 eV, and 288.1 eV, corresponding to carbon species from unsaturated hydrocarbons (sp^2^ hybridization, C=C bond), C–O bonds, and carbonyl groups, respectively [38]. Notably, FB2 exhibits a similar distribution of C 1s peaks as FB1 (Figure 8b vs. Figure 8a). These C 1s peaks indicate that the organic template, Pluronic P123, was degraded into unsaturated and oxidized species during the sol–gel synthesis process (Figure 3).

Next, FB3 exhibits a markedly different C 1s region compared to FB2 (Figure 8c vs. Figure 8b). The peaks observed at 284.8 eV, 286.1 eV, and 289.4 eV can be attributed to carbon species from saturated hydrocarbons (sp^3^ hybridization, C–C bond), C–O bonds, and carboxyl groups, respectively (Figure 8c). Given that the synthesis of FB3 did not involve calcination, in contrast to FB2 (refer to Figure 3), it is likely that a greater proportion of the original components of Pluronic P123 were retained in FB3. However, FB4 displays a C 1s region that is quite similar to that of FB3 (Figure 8d vs. Figure 8c), suggesting that the chemical valence of Fe ions had a minimal impact on the retention of organic residues in the catalyst.

**Figure 9:**
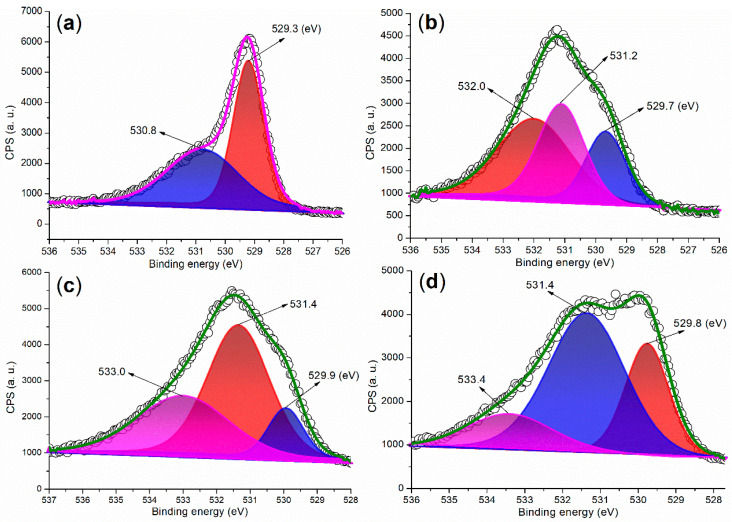
XPS measurements of the O 1s region: (**a**) FB1; (**b**) FB2; (**c**) FB3; (**d**) FB4.

The XPS analysis of the O 1s regions of the synthesized catalysts provides structural information from a different perspective. In the O 1s region of FB1, two peaks are observed at 529.3 eV and 530.8 eV (Figure 9a), which can be attributed to oxygen species associated with Fe (II or III)–O bonds [35] and organic residues, respectively. This finding aligns well with the results obtained from XRD (Figure 6a) and the Fe 2p region of the XPS analysis (Figure 5a).

When barium was introduced into the sol–gel synthesis (Figure 3), the O 1s regions of the synthesized catalysts became more complex (Figure 9b–d vs. Figure 9a). For instance, the first peak at 529.7 eV in the O 1s region of FB2 can be attributed to oxygen associated with Fe (II or III)–O bonds (Figure 9b) [35]. The subsequent peak at 531.2 eV appears to correspond to oxygen incorporated within the Fe–Ba mixed oxide lattice [36], while the last peak at 532.0 eV likely originates from oxygen in organic residues. FB3 and FB4 display similar curves with three O 1s peaks, akin to FB2 (Figure 9c,d vs. Figure 9b), indicating the presence of Fe (II or III)–O bonds, the mixed oxide lattice, and organic residues.

#### 2.2.2. Functional Group

**Figure 10:**
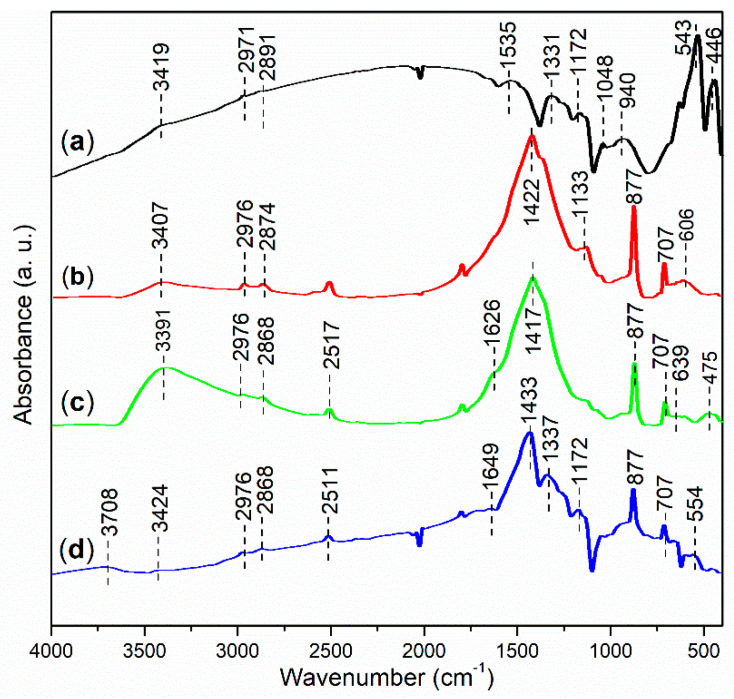
FT-IR spectra (adsorption mode) of synthesized catalysts: (**a**) FB1; (**b**) FB2; (**c**) FB3; (**d**) FB4.

FT-IR spectroscopy was performed to investigate the functional groups present in the synthesized catalysts. As shown in Figure 10a, the peak at 3419 cm^−1^ indicates the O–H stretching vibration of hydroxyl groups associated with iron oxides and organic residues on the surface of FB1 [39]. The subsequent peaks at 2971 cm^−1^ and 2891 cm^−1^ can be attributed to the anti-symmetric and symmetric stretching vibrations of C–H bonds in methyl groups [34], which likely originate from organic residues on FB1 (Figure 3). Additionally, FB1 exhibits a small peak at 1535 cm^−1^, probably corresponding to the C=C stretching of organic residues [40]. Furthermore, the peak at 1331 cm^−1^ is characteristic of the C–H bending vibration of methyl groups [41].

FB1 also displays a peak at 1172 cm^−1^, indicating the stretching of ether bonds (C–O–C) [42], primarily derived from Pluronic P123 (Figure 3). The subsequent peak at 1048 cm^−1^ is characteristic of C–O stretching in ester groups (O=C–O) [43], while the peak at 940 cm^−1^ corresponds to the C≡C bond of alkynes [44], likely representing organic residues remaining after calcination (Figure 3). Finally, the FT-IR peaks at 543 cm^−1^ and 446 cm^−1^ can both be attributed to the stretching vibrations of Fe–O bonds [45].

FB2 exhibits a fundamentally similar FT-IR response to FB1 in the wavenumber range of 4000–2500 cm^−1^ (Figure 10b vs. Figure 10a). However, FB2 features a distinct peak at 1422 cm^−1^, which is indicative of the C–C stretching vibrations [41], likely originating from organic residues remaining after calcination (Figure 3). Therefore, it seems that FB2 contains more organic residues than FB1, probably due to the incorporation of BaSO_4_ into the sol–gel synthesis (Figure 3). Additionally, the peak at 1133 cm^−1^ corresponds to the stretching of ether bonds (C–O–C), still derived from Pluronic P123 (Figure 3). The subsequent peaks at 877 cm^−1^ and 707 cm^−1^ are both characteristic of the bending vibrations of benzene skeletons, which may have formed after the calcination of the organic template (Pluronic P123) (Figure 3). Finally, the broad band centered at 606 cm^−1^ likely reflects the vibrations of metal–O bonds in FB2.

The FT-IR spectrum of FB3 exhibits a notable similarity to that of FB2 within the wavenumber range of 4000–400 cm^−1^ (Figure 10c vs. Figure 10b). However, FB3 is particularly characterized by a band centered at 3391 cm^−1^, which displays a significantly higher relative intensity compared to FB2 (Figure 10c vs. Figure 10b). This difference can be attributed to the absence of calcination at 550 °C during the synthesis of FB3, unlike FB2 (Figure 3). As a result, FB3 retains a greater amount of organic species containing hydroxyl groups, and meanwhile, a larger proportion of –OH units on the surface of the mixed oxide remains intact more than dehydration. In contrast, FB4 presents a FT-IR spectrum that closely resembles that of FB2 (Figure 10d vs. Figure 10b), further suggesting that the chemical valence of iron ions has minimal impact on the proliferation of functional groups in the synthesized catalysts (Figure 3).

**Figure 11:**
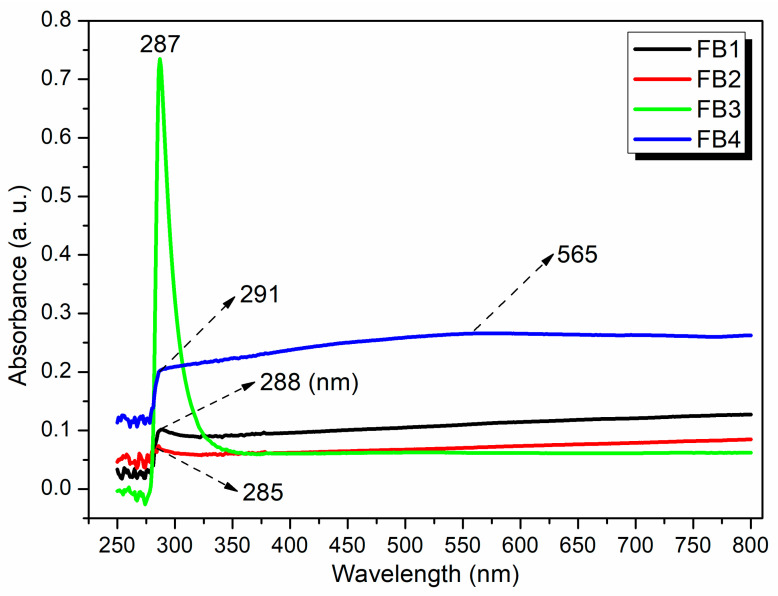
UV–vis spectra of synthesized catalysts FB1–FB4.

To further investigate the structures of the synthesized catalysts, UV–Vis spectroscopy was conducted after dispersing FB1–FB4 in water. All tested samples (FB1–FB4) exhibited adsorption in the range of 285–291 nm (Figure 11), which can be attributed to the charge transfer transition of organic residues within the synthesized catalysts [34]. Notably, FB3 displayed a sharp peak at 287 nm, with a relative intensity significantly higher than that of the other catalysts (green curve, Figure 11). This enhancement is primarily due to the lack of calcination at 550 °C for FB3, which allowed it to retain a greater quantity of organic species (Figure 3).

FB4 presented an additional broad band centered at 565 nm (blue curve, Figure 11), corresponding to the ligand-to-metal charge transfer (LMCT) transition of the metal species [34]. This observation suggests that utilizing Fe^2+^ salt as a raw material may facilitate a structure in which organic residues coordinate more effectively with the metal center in the catalyst (Figure 3).

#### 2.2.3. Thermal Stability

**Figure 12:**
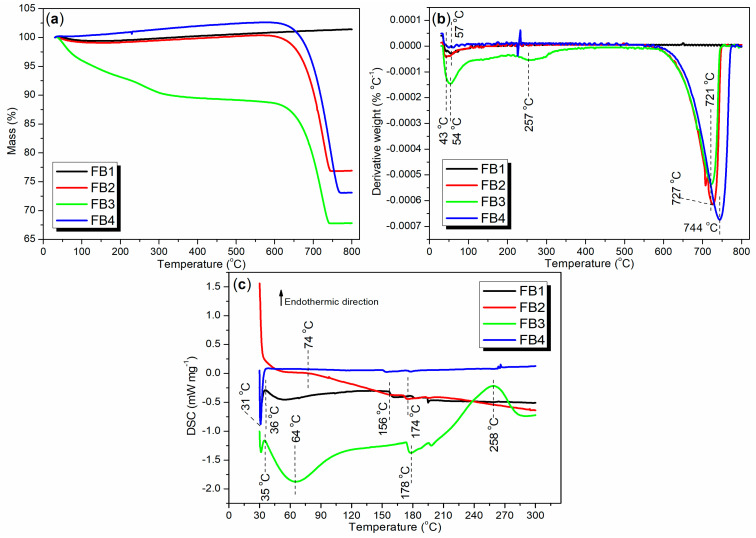
Thermal analysis of synthesized catalysts FB1–FB4: (**a**) TGA; (**b**) DTG; (**c**) DSC.

Thermogravimetric analysis (TGA and DTG), along with differential scanning calorimetry (DSC), can provide valuable insights into the composition and thermal stability of the synthesized catalysts. To evaluate the thermal stability of the catalysts under realistic production conditions, air flow was employed as the purge gas instead of nitrogen. As illustrated in Figure 12a, the TGA curve for FB1 initially shows a slight decline between 30 and 186 °C, with a weight loss of 0.55%, indicating the removal of adsorbed water or organic solvents. Subsequently, from 186 to 800 °C, the TGA curve exhibits a slight upward trend, accompanied by a weight increase of 1.95%, primarily due to the oxidation of the FeO component into Fe_2_O_3_ (equivalent to FeO_1.5_). Overall, the total weight increase of FB1 from 30 to 800 °C is 1.39% (black line, Figure 12a).

Correspondingly, only one distinct weight loss rate was observed at 57 °C, with no other significant weight loss rates detected between 57 and 800 °C (black line, Figure 12b). This indicates that the removal of adsorbed water or organic solvents occurs much more rapidly than the oxidation of FeO to Fe_2_O_3_. Additionally, FB1 exhibits its first endothermic peak at 36 °C, which corresponds to the energy required to remove the adsorbed water or organic solvents (black line, Figure 12c). A subsequent endothermic effect is observed between 50 and 156 °C, primarily reflecting the heat involved in the oxidation of FeO to Fe_2_O_3_ (black line, Figure 12c). Based on this analysis, it is evident that FB1 demonstrates considerable stability when heated up to 800 °C.

FB2 exhibits a TGA curve that closely resembles that of FB1 between 30 and 550 °C (red vs. black, Figure 12a), indicating a similar mechanism for the removal of adsorbed water and organic residues. However, beyond 550 °C, a significant and sharp weight loss of 23.45% is observed between 550 and 800 °C (red, Figure 12a). In contrast, FB1 shows no weight loss in this temperature range (black, Figure 12a). This weight loss in FB2 is likely due to the reduction of Ba-containing oxides, such as BaO_2_, by carbon (from organic residues) into BaO and CO_2_ [46].

Accordingly, two distinct weight loss rates are observed at 43 °C and 727 °C, corresponding to the two weight loss events (red line of Figure 12b vs. red line of Figure 12a). Concurrently, the DSC curve for FB2 reveals two continuous exothermic effects: the first occurring between 30 °C and 74 °C, and the second between 74 °C and 300 °C (red, Figure 12c). This suggests that the removal of adsorbed water and organic residues from FB2 is, in fact, an exothermic process.

The lack of calcination at 550 °C during synthesis (Figure 3) likely contributes to the behavior observed in FB3. Initially, FB3 exhibits a significant and continuous weight loss of 11.39% between 30 °C and 600 °C (green, Figure 12a), indicating the release of adsorbed water and organic residues upon heating. Following this, a further weight loss of 20.80% occurs from 600 °C to 800 °C, which is associated with the reduction of Ba-containing oxides (green, Figure 12a).

Additionally, three distinct weight loss rates are identified at 54 °C, 257 °C, and 721 °C (green, Figure 12b). The first two rates correspond to the removal of adsorbed water and organic residues, while the last rate reflects the reduction of Ba-containing oxides. Furthermore, FB3 displays two notable endothermic peaks at 35 °C and 258 °C, along with two exothermic peaks at 64 °C and 178 °C (green, Figure 12c). These thermal events illustrate the energy dynamics involved in the removal of adsorbed water and organic residues.

The TGA and DTG curves of FB4 exhibit similar trends to those of FB2, despite differences in exact values (blue vs. red, Figure 12a,b). In terms of DSC analysis, a sharp exothermic peak at 31 °C likely indicates the evaporation of volatile organics in FB4, which releases heat. Notably, there are no additional thermal effects observed between 38 °C and 300 °C (blue, Figure 12c).

#### 2.2.4. Morphology and Energy-Dispersive Spectroscopy

**Figure 13:**
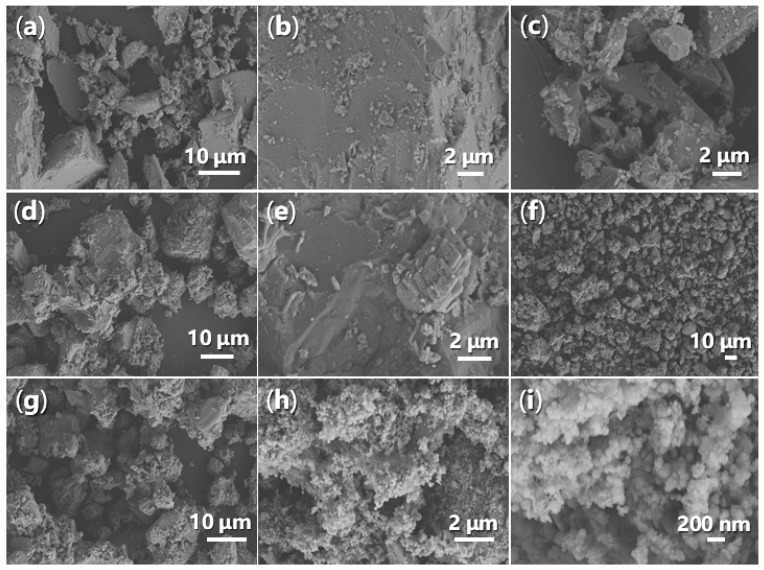
SEM images of synthesized catalysts: (**a**) FB1 (magnification 1770×); (**b**) FB1 (5280×); (**c**) FB1 (6640×); (**d**) FB2 (1400×); (**e**) FB2 (7440×); (**f**) FB3 (396×); (**g**) FB3 (1770×); (**h**) FB4 (8780×); (**i**) FB4 (39,240×).

It is crucial to examine the morphology of synthesized catalysts using SEM, as this can provide valuable insights into the stability of the catalysts during catalysis. Initially, FB1 consists of large blocks measuring 1–15 μm, alongside smaller particles ranging from 100 to 500 nm (Figure 13a,c). The surface of the larger particles is generally smooth with sharp edges (Figure 13b). However, upon the addition of BaSO_4_ as a dopant (Figure 3), the resulting FB2 exhibits a fluffy texture, with rounded edges and corners (Figure 13d,e).

Furthermore, in the absence of calcination during synthesis, the resulting catalyst (FB3) exhibits a markedly different morphology compared to FB2 (Figure 13f,g vs. Figure 13d,e). All particles of FB3, ranging in size from 5 to 30 μm, display a porous texture (Figure 13f,g), likely indicating that most of the organic template (Pluronic P123, Figure 3) was retained in the synthesized sample. Conversely, when Fe^2+^ salt was used as the raw material instead of Fe^3+^ salt (Figure 3), the resulting FB4 manifested as an agglomeration of particles with an average diameter of 100 nm (Figure 13h,i), suggesting a difference in crystallinity. This morphology of FB4 also leads to the highest zeta potential of its aqueous particles among all synthesized catalysts (Table 1), probably meaning the best stability in catalysis.

**Figure 14:**
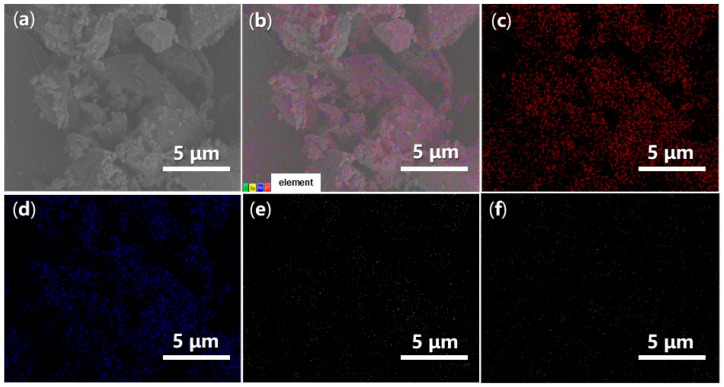
SEM and EDS mapping images of FB1: (**a**) SEM of FB1 (magnification 6640×, same as Figure 13c with different scale bar); (**b**) EDS layered image; (**c**) O Kα1; (**d**) Fe Lα1,2; (**e**) Ba Mα; (**f**) S Kα1.

**Figure 15:**
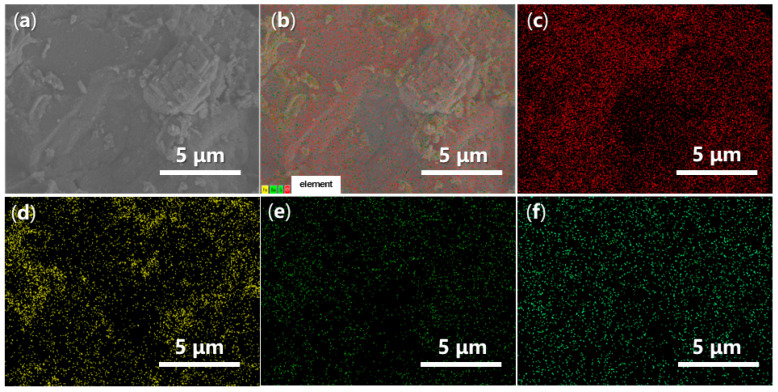
SEM and EDS mapping images of FB2: (**a**) SEM of FB2 (magnification 7440×, same as Figure 13e with different scale bar); (**b**) EDS layered image; (**c**) O Kα1; (**d**) Fe Lα1,2; (**e**) Ba Mα; (**f**) S Kα1.

Energy-Dispersive Spectroscopy (EDS) can provide valuable insights into the elemental dispersion across the synthesized catalysts. As illustrated in Figure 14, both O and Fe are evenly distributed throughout FB1 (Figure 14c,d vs. Figure 14a,b), while the presence of Ba and S is significantly limited, indicating contamination (Figure 14e,f vs. Figure 14c,d). In contrast, when BaSO_4_ was introduced as a raw material (Figure 3), the resulting FB2 exhibited a comprehensive elemental dispersion of O, Fe, Ba, and S (Figure 15).

### 2.3. Catalytic Chlorination

#### 2.3.1. Effects of Catalysts

First, when the combination of HCl and H_2_O_2_ was utilized as the sole chlorine source without any catalysts, the chlorination of toluene resulted in a moderate conversion of 64%. The molar ratio of 2-chlorotoluene to 4-chlorotoluene was found to be 2.55:1 (entry 1, Table 2). This observation supports the previously reported mechanism, which posits that the combined use of HCl and H_2_O_2_ mimics haloperoxidase enzymes, facilitating chlorination through the oxidation of Cl^−^ by H_2_O_2_ (Figure 2c) [18]. Additionally, the chloro group acts as an *ortho–para*-directing group, enhancing the stereoselectivity of the chlorinated product [47].

Second, the introduction of synthesized catalysts such as FB1 and FB2 significantly enhances chlorination conversions compared to the catalyst-free experiment (entries 4–5 vs. 1, Table 2). This suggests that the oxide structures of FB1 and FB2 may facilitate the oxidation of Cl^−^ by H_2_O_2_ (Figure 2c), thereby improving the utilization rate of Cl^−^. In contrast, FB3 exhibited a much lower chlorination conversion than the catalyst-free experiment (entry 6 vs. 1, Table 2). This is primarily due to the absence of calcination (at 550 °C) during the synthesis of FB3, which resulted in a substantial amount of organic residues remaining in the catalyst. These residues may inhibit the oxidation of Cl^−^ by H_2_O_2_ by reducing Cl^+^.

Third, FB1 and FB2 exhibit molar ratios of *ortho*-chlorinated and *para*-chlorinated products of 3.64:1 and 2.15:1, respectively (entries 4–5, Table 2). The former ratio is higher than that obtained from the catalyst-free experiment (entry 4 vs. 1, Table 2), while the latter is lower (entry 5 vs. 1, Table 2). This indicates that the incorporation of Ba into the catalyst increases the overall conversion of chlorination but decreases stereoselectivity (Figure 3; entries 5 vs. 4, Table 2).

Next, FB4 demonstrates lower total conversion and *ortho–para* stereoselectivity compared to both the catalyst-free experiment and FB2 (entries 7 vs. 1 and 5). This is attributed to FB4 containing a higher concentration of Fe^2+^-containing components than FB2, as indicated by the Fe 2p region of FB4 extending into the binding energy range below 710 eV, unlike that of FB2 (Figure 5d vs. Figure 5b). Consequently, it appears that Fe^2+^-containing components have a detrimental effect on both chlorination conversion and stereoselectivity.

On the other hand, when benzene was used as the substrate, the catalyst-free experiment achieved an 83% conversion with a corresponding yield of chlorobenzene, without producing any by-products (entry 1, Table 3). However, the use of synthesized catalysts significantly improved chlorination conversions (entries 2–7 vs. 1, Table 3). FB3 appeared to be less effective than the other catalysts (entries 4 vs. 2, 3, and 5, Table 3), suggesting that the organic residues in FB3 have the most pronounced negative impact on catalyst activity, primarily due to the absence of calcination during its synthesis (Figure 3).

Additionally, when *tert*-butyl hydroperoxide (TBHP) was used as the substrate, the corresponding chlorination did not require H_2_O_2_ as an initiator (Table 4). TBHP exhibits strong oxidation activity, which may facilitate the oxidation of Cl^−^ into ClO^−^, similar to H_2_O_2_ [48]. Furthermore, FB2 produced significantly more di-chlorinated and tri-chlorinated products than FB1 (entries 1 vs. 2, Table 4). This aligns with the higher conversions achieved by FB2 compared to FB1 in the chlorination of both toluene (entries 5 vs. 4, Table 2) and benzene (entries 3 vs. 2, Table 3). Thus, it can be concluded that the incorporation of Ba into the catalyst enhances chlorination efficiency, likely because Ba^2+^-containing components may catalyze the oxidation of Cl^−^ into Cl^+^ using either H_2_O_2_ or TBHP.

#### 2.3.2. Effects of Catalyst and Hydrogen Chloride Dosages

It is also interesting to investigate the effects of catalyst dosage on catalytic chlorination. When the loading amount of FB2 was doubled in the catalytic chlorination of toluene, the conversion increased from 82% to 100%, and the molar ratio of 2-chlorotoluene to 4-chlorotoluene improved from 2.15 to 2.70 (entries 9 vs. 5, Table 2). Meanwhile, when benzene was used as the substrate, FB1 exhibited a higher conversion when its loading amount was doubled (entries 6 vs. 2, Table 3). Notably, there was no decrease in conversion when the loading amount of FB2 was also doubled (entries 7 vs. 3, Table 3). Therefore, it can be concluded that in the catalytic chlorination reactions of toluene and benzene, an increase in catalyst dosage allows for a more efficient transformation of active intermediates like Cl⁺ into products, minimizing the formation of undesired by-products.

On the other hand, FB2 demonstrated a significant increase in the conversion of toluene when the loading amount of HCl was doubled (entries 8 vs. 5, Table 2). Concurrently, the molar ratio of 2-chlorotoluene to 4-chlorotoluene increased from 2.15 to 2.33 (entries 8 vs. 5, Table 2). This suggests that a higher concentration of Cl^−^ around the catalyst likely leads to the generation of more ClO^−^, thereby enhancing the overall conversion.

#### 2.3.3. Effects of Oxidants

The oxidants used in this study warrant attention, as they play a crucial role in facilitating chlorination. Initially, in the absence of H_2_O_2_ as an oxidant, FB2 produced no chlorinated products at both 60 °C and 80 °C (entries 2–3, Table 2). This result indicates that the synthesized catalyst, FB2, is unable to oxidize Cl^−^ into ClO^−^ without the assistance of H_2_O_2_.

Considering the oxidation properties of H_2_O_2_, another oxidant, peracetic acid, was employed. This also facilitated the chlorination of benzyl alcohol, similar to H_2_O_2_ (entries 2 vs. 1, Table 5). However, both the oxidized product (benzaldehyde, compound **D**, Table 5) and the dehydrated product (1-((benzyloxy)methyl)benzene, compound **E**, Table 5) were produced simultaneously (entry 2, Table 5). This result further underscores that the oxidation of Cl^−^ into ClO^−^ is a critical step in the chlorination process, which can be achieved using various oxidants.

#### 2.3.4. Effects of Substrates

The synthesized catalysts, particularly FB2, demonstrate excellent catalytic properties in the chlorination of toluene (Table 2), benzene (Table 3), and TBHP (Table 4). Moderate yields were achieved in the chlorination of benzyl alcohol (Table 5), while the chlorination of ethylbenzene, *tert*-butylbenzene, acetophenone, *N*,*N*-dimethylbenzenamine, bromobenzene, and iodobenzene resulted in moderate to poor outcomes (Table 6).

Due to the steric hindrance of alkyl substituents such as ethyl and *tert*-butyl, *para*-chlorinated arenes emerged as the dominant products over *ortho*-chlorinated ones (entries 1–2, Table 6). When acetophenone was used as the substrate, chlorination occurred preferentially on the methyl group rather than the benzene ring (entry 3, Table 6). This is likely because the acetyl group has a strong electron-withdrawing effect, which tightly attracts the π electron cloud, making an electrophilic attack by ClO^−^ on the benzene ring quite difficult. Furthermore, when the substrate was changed to *N*,*N*-dimethylbenzenamine, the *ortho*-chlorinated product surpassed the *para*-chlorinated one (entry 4, Table 6) due to the directing effects of the dimethylamino group.

Notably, bromobenzene can be chlorinated using the present protocol, resulting exclusively in chlorobenzene (entry 5, Table 6), which, to the best of our knowledge, has not been previously reported. Additionally, when iodobenzene was utilized as the substrate, chlorobenzene, 1-chloro-4-iodobenzene, and 1-chloro-2-iodobenzene were produced (entry 6, Table 6). This indicates that the present catalyst (FB2) is capable of not only chlorinating the C–H bonds of the benzene ring in iodobenzene but also substituting the iodine group with a chlorine atom.

### 2.4. Proposed Process for Catalytic Chlorination

**Figure 16:**
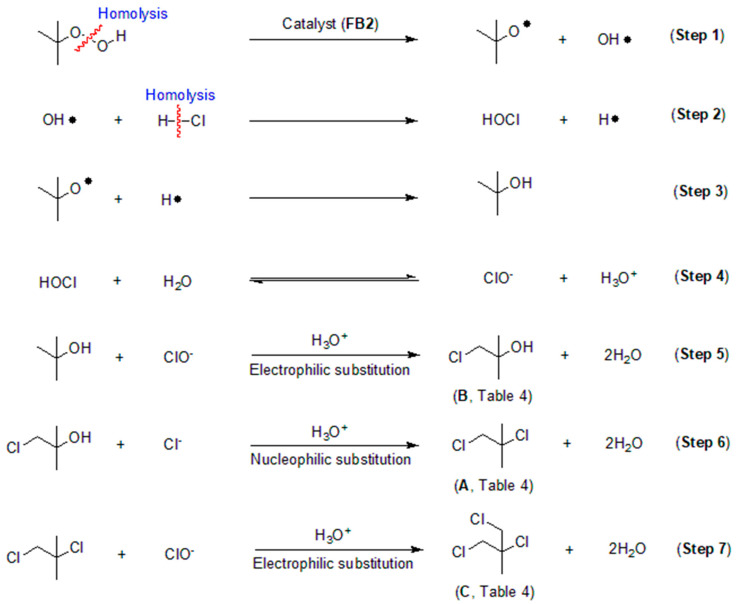
Proposed process for catalytic chlorination of *tert*-butyl hydroperoxide (TBHP).

Based on the data obtained thus far, it is intriguing to explore the process behind the catalytic chlorination of TBHP, as illustrated in Table 4. It appears that TBHP functions as both an oxidant (for its oxidation of HCl into HClO) and a substrate in the chlorination process. Initially, TBHP may undergo homolysis in the presence of FB2, resulting in the formation of a *tert*-butoxy radical and a hydroxyl radical (OH·), as depicted in Step 1 of Figure 16. Previous studies have confirmed that such homolysis can occur with iron(III) porphyrin complexes [49]. Subsequently, the OH· radical can react with HCl, producing hypochlorous acid (HOCl) and a hydrogen free radical (H·), as shown in Step 2 of Figure 16. The H· radical can then combine with the *tert*-butoxy radical, yielding *tert*-butyl alcohol, as illustrated in Step 3 of Figure 16.

HOCl can be hydrolyzed to generate ClO^−^, which subsequently undergoes electrophilic substitution with *tert*-butyl alcohol, resulting in the formation of the mono-chlorinated product (compound **B**, Table 4; Steps 4–5, Figure 16). Compound **B** can further react with Cl^−^ via nucleophilic addition to produce compound **A** (Table 4), the di-chlorinated product (Step 6, Figure 16). Finally, compound **A** can react with ClO^−^ through another electrophilic substitution, yielding the tri-chlorinated product **C** (Table 4; Step 7, Figure 16).

## 3. Experimental Section

### 3.1. Starting Materials

Pluronic P123 (EO_20_–PO_70_–EO_20_; EO, ethylene oxide; PO, propylene oxide; average *M*_n_, 5800), Fe(NO_3_)_3_·9H_2_O (iron(III) nitrate nonahydrate, 99%), BaSO_4_ (barium sulfate, 2 μm, 99%), FeSO_4_·7H_2_O (iron(II) sulfate heptahydrate, 99%), NH_3_·H_2_O (ammonium hydroxide solution, 25%), H_2_O_2_ (hydrogen peroxide solution, 30% in H_2_O), and HCl (hydrochloric acid, 37% in H_2_O) were bought from Shanghai Aladdin Biochemical Technology Co., Ltd., Shanghai, China. Peracetic acid solution (C_2_H_4_O_3_, 35% in acetic acid) was commercially available from Tianjin Fuyu Fine Chemical Co., Ltd., Tianjin, China. Toluene (99%), benzene (99%), TBHP (*tert*-butyl hydroperoxide solution, 70% in H_2_O), benzyl alcohol (99%), ethylbenzene (99%), *tert*-butylbenzene (99%), acetophenone (99%), bromobenzene (99%), and iodobenzene (98%) were all purchased from Shanghai Macklin Biochemical Technology Co., Ltd., Shanghai, China. *N*,*N*-dimethylbenzenamine was commercially available from Chengdu Kelong Chemical Reagent Factory, Chengdu, China.

### 3.2. Analytical Instruments

X-ray photoelectron spectroscopy (XPS) was conducted using a Kratos Axis Ultra DLD (Kratos Co., Ltd., Manchester, UK), with monochromatic Al Kα X-rays (1486.6 eV) as the excitation source. The binding energy scale was calibrated using the C 1s peak at 284.8 eV as the standard reference. Peak fitting was performed using a Gaussian–Lorentzian (G/L) product function with a 30% Lorentzian ratio.

Wide-angle X-ray diffraction (XRD, 2*θ* = 10–80°) measurements were carried out to assess the crystallinity of the synthesized catalyst. This was performed on a Philips X’Pert Pro diffractometer (PANalytical B.V. Co., Ltd., Almelo, Netherlands), utilizing Cu Kα radiation (λ = 1.5418 Å) with a scanning interval of 0.05° s^−1^ over a range of 2*θ* = 10–80°.

Zeta (ζ) potential was measured using a Zetasizer Nano ZSE instrument manufactured by Malvern, United Kingdom. This instrument has a particle size test range of 0.30 nm to 10 μm and operates at a test angle of 175° ± 12.8°. It features a high-speed digital correlator with over 4000 physical channels and a linear range exceeding 10^11^. The detection position can continuously move between 0.45 mm and 4.65 mm from the pool wall, allowing for concentration measurements at a single angle. The mobility measurement range is nominally ±10 μcm Vs^−1^, while the conductivity measurement range spans from 0 to 200 mS cm^−1^.

FT-IR spectra were obtained using a VERTEX 70 instrument manufactured by Bruker, Berlin, Germany. This instrument has a spectral range of 400 to 4000 cm^−1^. It offers a wavenumber accuracy of 0.02 cm^−1^ and a spectral resolution better than 0.4 cm^−1^, which is continuously adjustable. The minimum step size is 0.1 cm^−1^, and the infrared host signal-to-noise ratio (peak-to-peak) exceeds 50,000:1. UV–Vis spectra were acquired using a Lambda 950 instrument manufactured by PerkinElmer, United States. This instrument has a wavelength range of 190 to 3300 nm and exhibits a stray light level of approximately ≤ 0.00007%T.

Both thermogravimetric analysis (TGA) and derivative thermogravimetry (DTG) were conducted using a METTLER instrument manufactured by METTLER TOLEDO, Switzerland. The temperature range for these analyses extends from 30 °C to 800 °C, with a temperature accuracy of ±0.3 °C. The balance sensitivity is 0.1 μg, and the balance measurement accuracy is 0.005%. The instrument features built-in weights for automatic calibration, a heating rate 10 °C min^−1^, and a furnace cooling time of 20 min (from 800 °C to 100 °C). The experimental atmosphere used air.

Differential scanning calorimetry (DSC) was performed using the METTLER TOLEDO DSC 3 instrument, manufactured by METTLER TOLEDO, Switzerland. This instrument is equipped with FRS 5+ and HSS 8+ sensors and has a temperature range of −170 to 700 °C, with a temperature accuracy of ± 0.1 °C. The heating rate is 10 °C min^−1^. The calorimetric sensitivity is 0.04 μW for the FRS 5+ sensor (Professional).

SEM and EDS-SEM images were acquired using the GeminiSEM 500 instrument manufactured by Carl Zeiss (Shanghai) Management Co., Ltd., China. This instrument is equipped with energy spectrum and super energy spectrum capabilities, offering a resolution of approximately 0.6 nm at 15 kV and 0.9 nm at 1 kV (with the series deceleration option). The acceleration voltage is 15 kV, and it provides a magnification range of ×20 to ×2,000,000. The probe current ranges from 3 pA to 20 nA.

GC-MS analyses were conducted using the GC-MS-QP2010 Plus from Shimadzu, equipped with an Rxi-5ms capillary column measuring 30 m in length and 0.25 mm in internal diameter. For the gas chromatography (GC) portion, the column temperature was set to 60 °C, and the injection port temperature was maintained at 250 °C. The sampling mode employed was split-flow, with a split ratio of 26, and helium was selected as the carrier gas. In the mass spectrometry (MS) section, the ion source temperature was set to 200 °C, while the interface temperature was maintained at 250 °C.

### 3.3. Synthesis of Catalysts

As illustrated in Figure 3, Pluronic P123 (0.58 g, 0.1 mmol) is combined with 50 mL of distilled water in a 100 mL round-bottom flask, while being stirred magnetically at 20 °C. Stirring is continued until the solution becomes clear, resulting in Solution A. Next, Fe(NO_3_)_3_·9H_2_O (0.404 g, 1 mmol for the synthesis of FB1–FB3) or FeSO_4_·7H_2_O (0.278 g, 1 mmol for the synthesis of FB4) is added as the iron source. Immediately thereafter, BaSO_4_ is introduced as a supporting material (none for FB1; 0.233 g, 1 mmol for FB2–FB4). The resulting mixture is stirred further at 20 °C for 20 min, yielding Solution B.

Subsequently, 10 mL of 25% NH_3_·H_2_O is added, and the resulting suspension is stirred at 20 °C for an additional 30 min. The reddish-brown solid is then separated by filtration under reduced pressure and washed three times with 20 mL of distilled water and 20 mL of absolute ethanol, respectively. The collected solid is dried in a baking oven at 80 °C for 3 h, followed by calcination at 550 °C in a muffle oven (calcination is performed for the synthesis of FB1, FB2, and FB4; for FB3, calcination is omitted). This process yields the synthesized catalysts (FB1–FB4) utilized in this study.

### 3.4. Catalytic Chlorination

The synthesized catalysts (FB1–FB4, with 0–4 mol% Fe relative to the substrate) and the substrate (1.0 mmol) were combined with aqueous HCl (37 wt.%, 4–8 mmol pure HCl) in a 250 mL three-necked round-bottom flask equipped with a condenser and an oil bath, all maintained at room temperature. Under magnetic stirring, the initiator (either H_2_O_2_ or peracetic acid, 0.5 mmol; for the chlorination of TBHP, none, as indicated in Table 4) was added dropwise via an addition funnel. The temperature was then raised to 60–80 °C while continuing to stir magnetically, and the reaction mixture was allowed to react at this temperature for 6 h. After the reaction was complete, the mixture was filtered through filter paper, and the resulting filtrate was analyzed using GC-MS to determine the chlorination conversion and the yield of the isolated product.

## 4. Conclusions

In this study, a series of Fe–Ba mixed oxides were synthesized via a sol–gel process, utilizing Pluronic P123 as a template. These materials were thoroughly characterized and subsequently employed as catalysts for the chlorination of various organic molecules, using commercial hydrochloric acid as the chlorine source and aqueous hydrogen peroxide as the oxidant. The results are summarized as follows.

(1) The synthesized oxide catalyst, composed solely of Fe, primarily consists of Fe_2_O_3_, with a small proportion of Fe_3_O_4_. This catalyst demonstrates excellent thermal stability when heated from 30 °C to 800 °C and exhibits moderate to high conversions in the chlorination of toluene, benzene, and *tert*-butyl hydroperoxide. Notably, this catalyst displays the highest *ortho*-selectivity among the Fe–Ba mixed oxide catalysts in the chlorination of toluene.
(2) The incorporation of Fe^3+^ salt with BaSO_4_ in the sol–gel process, followed by calcination at 550 °C, yields a Fe–Ba mixed oxide catalyst composed of BaO_2_, BaFe_4_O_7_, and Fe_2_O_3_. This catalyst experiences weight loss when heated to 600 °C or higher, attributed to the thermal reduction of BaO_2_. It demonstrates significantly higher conversions compared to the pure Fe-containing catalyst in the chlorination of toluene and benzene. Notably, this catalyst produces a tri-chlorinated product compared to the pure Fe-containing catalyst in the chlorination of *tert*-butyl hydroperoxide (TBHP).
(3) Other Fe–Ba mixed oxide catalysts, including those that do not undergo calcination at 550 °C and those that utilize Fe^2+^ salt as the raw material in the sol–gel synthesis, exhibit comparatively lower chlorination activity. This reduced performance is likely due to the presence of organic residues in the catalysts, as well as the excessive Fe^2+^-containing components.
(4) The chlorination of *tert*-butyl hydroperoxide (TBHP) does not require additional oxidants such as H_2_O_2_ or peracetic acid, as TBHP itself serves as both the substrate and the oxidant during the reaction. This process encompasses both electrophilic and nucleophilic substitutions within the catalytic process.
(5) The chlorination of bromobenzene yields chlorobenzene as the sole product, with a yield of 18%. Meanwhile, the chlorination of iodobenzene produces chlorobenzene, 1-chloro-4-iodobenzene, and 1-chloro-2-iodobenzene. To the best of our knowledge, these two reactions have not been previously reported, marking a significant advancement in the field of chlorination and its industrial applications.

Therefore, given the availability of the catalyst, the diversity of substrates, and the comparative safety and cleanliness of the chlorine source and oxidant, the current chlorination system is poised to significantly advance both the chlorination industry and related pharmaceutical developments.

## Figures and Tables

**Table 1:** Binding energy and atomic composition on surfaces of synthesized catalysts (depth, 0–3 nm) as well as zeta potential of aqueous catalyst particles.

Catalyst	C (1s)	O (1s)	Fe (2p)	Ba (3d)	*ζ* (mV) ^a^
FB1	283.80 (29.49) ^b^	528.80 (56.38)	709.80 (14.13)	- ^c^	0.0531
FB2	284.80 (46.23)	530.80 (51.26)	710.80 (2.49)	782.80 (0.02)	−13.3000
FB3	284.80 (45.07)	530.80 (47.28)	710.80 (7.38)	782.80 (0.26)	−13.1000
FB4	284.80 (42.86)	531.80 (48.51)	711.80 (7.90)	782.80 (0.73)	0.0070

^a^ Zeta potential of sample particle in H_2_O (mV). ^b^ Binding energy (eV), along with atomic percentage (at%) in parentheses. ^c^ Not founded or not counted by instrument due to low content.

**Table 2:** Catalytic C–H chlorination of toluene.

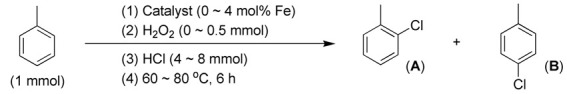							
Entry ^a^	Catalyst	Cat. Loading (mol% Fe) ^c^	H_2_O_2_ (mmol)	HCl (mmol)	T (°C)	Conversion (%) ^b^	Yield (%) ^c^
1	blank	0	0.5	4	60	64	46 (**A**), 18 (**B**)
2	FB2	2	0	4	60	0	0
3	FB2	2	0	4	80	0	0
4	FB1	2	0.5	4	60	79	62 (**A**), 17 (**B**)
5	FB2	2	0.5	4	60	82	56 (**A**), 26 (**B**)
6	FB3	2	0.5	4	60	36	19 (**A**), 17 (**B**)
7	FB4	2	0.5	4	60	63	32 (**A**), 31 (**B**)
8	FB2	2	0.5	8	60	100	70 (**A**), 30 (**B**)
9	FB2	4	0.5	4	60	100	73 (**A**), 27 (**B**)

^a^ Reaction conditions: toluene (1.0 mmol), catalyst (0–4 mol% Fe over toluene), H_2_O_2_ (aqueous, 30 wt.%, 0–0.5 mmol pure H_2_O_2_), HCl (aqueous, 37 wt.%, 4–8 mmol pure HCl), 60–80 °C, 6 h. ^b^ Conversion of toluene (%), determined by GC-MS (Figures S1–S4, Section S1, Supplementary Materials). ^c^ Yields (%) of isolated products, determined by GC-MS (Figures S1–S4, Section S1, Supplementary Materials).

**Table 3:** Catalytic C–H chlorination of benzene.

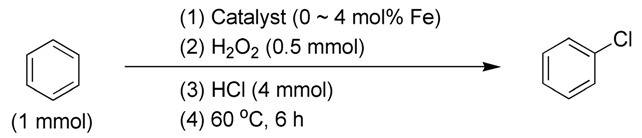				
Entry ^a^	Catalyst	Cat. Loading (mol% Fe)	Conversion (%) ^b^	Yield (%) ^c^
1	blank	0	83	83
2	FB1	2	98	98
3	FB2	2	100	100
4	FB3	2	90	90
5	FB4	2	100	100
6	FB1	4	100	100
7	FB2	4	100	100

^a^ Reaction conditions: benzene (1.0 mmol), catalyst (0–4 mol% Fe over benzene), H_2_O_2_ (aqueous, 30 wt.%, 0.5 mmol pure H_2_O_2_), HCl (aqueous, 37 wt.%, 4 mmol pure HCl), 60 °C, 6 h. ^b^ Conversion of benzene (%), determined by GC-MS (Figures S5 and S6, Section S2, Supplementary Materials). ^c^ Yield (%) of chlorobenzene, determined by GC-MS (Figures S5 and S6, Section S2, Supplementary Materials).

**Table 4:** The catalytic chlorination of *tert*-butyl hydroperoxide (TBHP).

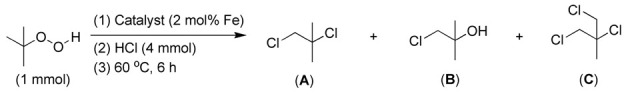			
Entry ^a^	Catalyst ^b^	Conversion (%) ^c^	Yield (%) ^d^
1	FB2	100	62 (**A**), 27 (**B**), 11 (**C**)
2	FB1	100	49 (**A**), 51 (**B**)

^a^ Reaction conditions: TBHP (1.0 mmol), catalyst (2 mol% Fe over TBHP), HCl (aqueous, 37 wt.%, 4 mmol pure HCl), 60 °C, 6 h. ^b^ Conversion of TBHP (%), determined by GC-MS (Figure S7, Section S3, Supplementary Materials). ^c^ Yields (%) of chlorinated products, determined by GC-MS (Figure S7, Section S3, Supplementary Materials).

**Table 5:** The chlorination of benzyl alcohol catalyzed by FB2 using different oxidants.

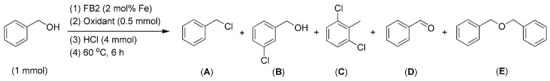			
Entry ^a^	Initiator	Conversion (%) ^b^	Yield (%) ^c^
1	H_2_O_2_	70	45 (**A**), 14 (**B**), 11 (**C**)
2	CH_3_(C=O)OOH	59	45 (**A**), 2 (**D**), 12 (**E**)

^a^ Reaction conditions: benzyl alcohol (1.0 mmol), catalyst (2 mol% Fe over benzyl alcohol), oxidant (H_2_O_2_ or CH_3_(C=O)OOH, 0.5 mmol), HCl (aqueous, 37 wt.%, 4 mmol pure HCl), 60 °C, 6 h. ^b^ Conversion of benzyl alcohol (%), determined by GC-MS (Figures S8 and S9, Section S4, Supplementary Materials). ^c^ Yields (%) of chlorinated products, determined by GC-MS (Figures S8 and S9, Section S4, Supplementary Materials).

**Table 6:** The chlorination of ethylbenzene, *tert*-butylbenzene, acetophenone, *N*,*N*-dimethylbenzenamine, bromobenzene, and iodobenzene catalyzed by FB2.

Entry ^a^	Substrate	Conversion (%) ^b^	Yield (%) ^c^
1	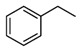	4	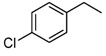 3 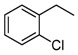 1
2	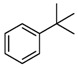	21	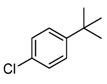 21
3	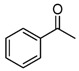	7	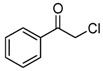 7
4	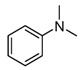	34	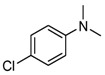 13 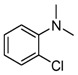 21
5	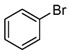	18	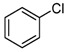 18
6	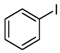	5	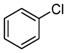 2.5 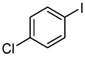 1.5 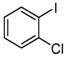 1

^a^ Reaction conditions: substrate (1.0 mmol), catalyst (2 mol% Fe over substrate), H_2_O_2_ (0.5 mmol), HCl (aqueous, 37 wt.%, 4 mmol pure HCl), 60 °C, 6 h. ^b^ Conversion of substrate (%), determined by GC-MS (Figures S10–S13, Section S5, Supplementary Materials). ^c^ Yields (%) of chlorinated products, determined by GC-MS (Figures S10–S13, Section S5, Supplementary Materials).

## Data Availability

The raw data supporting the conclusions of this article will be made available by the authors upon request.

## Supplementary Materials

The following supporting information can be downloaded at: https://www.mdpi.com/article/10.3390/molecules29225451/s1, Section S1: GC-MS examples for Table 2 (Figures S1–S4); Section S2: GC-MS examples for Table 3 (Figures S5 and S6); Section S3: GC-MS examples for Table 4 (Figures S7); Section S4: GC-MS examples for Table 5 (Figures S8 and S9); Section S5: GC-MS examples for Table 6 (Figures S10–S13).
