# Supplementary material for: The Efficient and Environmentally Friendly Chlorination of Arene, Alcohol, Halobenzene, and Peroxide Catalyzed by Fe–Ba Binary Oxides Using Hydrochloric Acid as Chlorine Source and Aqueous H2O2 as Oxidant
Source: Molecules. 2024 Nov 19;29(22):5451. doi: 10.3390/molecules29225451 (PMC11597577; doi:10.3390/molecules29225451)

Supplementary Materials

The Efficient and Environmentally Friendly Chlorination of Arene, Alcohol, Halobenzene, and Peroxide Catalyzed by Fe–Ba Binary Oxides Using Hydrochloric Acid as Chlorine Source and Aqueous H_2O_2 as Oxidant

Sidra Chaudhary [1], Qin Pan [1], Yong Wu [1,2], Zainab Bibi [1], Xiaoyong Li [1,2], Qinxiang Jia [1,2], Yang Sun [1,2,*]

[1] Department of Applied Chemistry, School of Chemistry, Xi'an Jiaotong University, No. 28, Xianning West Road, Xi'an 710049, P.R. China

[2] Xi'an Biomass Green Catalysis and Advanced Valorization International Science and Technology Cooperation Base, No. 28, Xianning West Road, Xi'an 710049, P.R. China

Table of Contents

S1. GC-MS examples for Table 2 (Figures S1–S4)
S2. GC-MS examples for Table 3 (Figures S5–S6)
S3. GC-MS examples for Table 4 (Figures S7)
S4. GC-MS examples for Table 5 (Figures S8–S9)
S5. GC-MS examples for Table 6 (Figures S10–S13)

S1. GC-MS examples for Table 2

* Corresponding author. Tel.: +86 29 82663914 (Y. Sun); fax: +86 29 82668559 (Y. Sun).
E-mail address: sunyang79@mail.xjtu.edu.cn (Y. Sun).

(1) Entry 1, Table 2

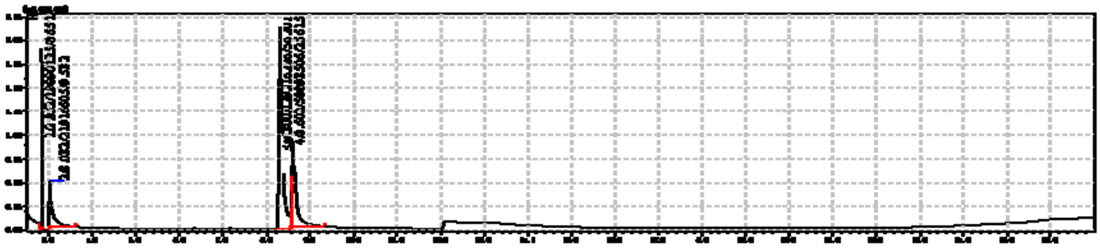

Figure S1. GC part of GC-MS for Entry 2, Table 2.

The peaks for t_R = 2.852 min and 3.032 are both:

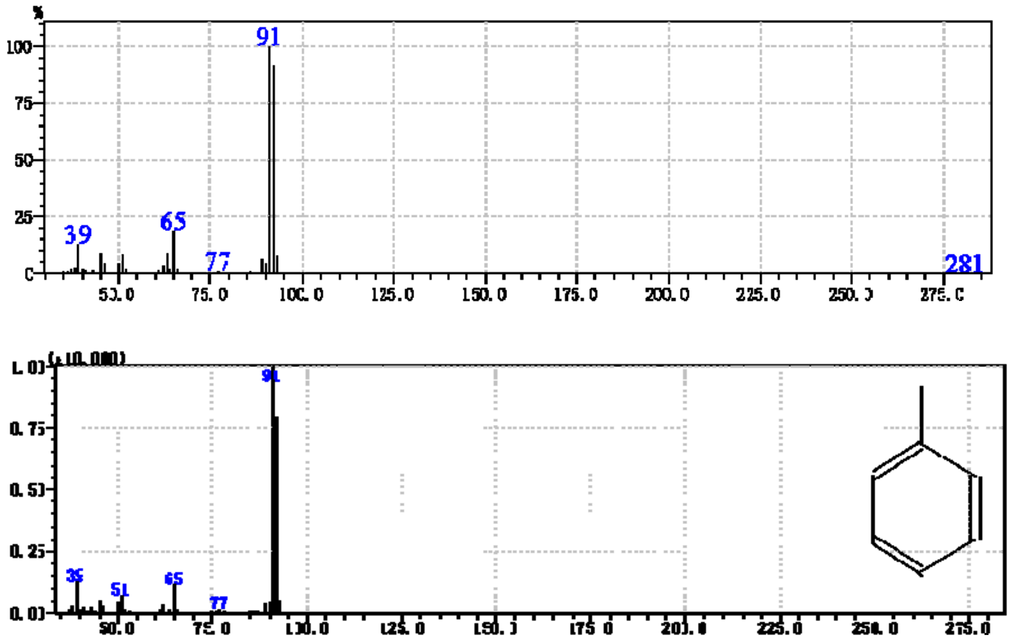

GC-MS: calcd. for C_7H_8 92, found 92 (C_7H_8). Table 2. Upper is the real mass spectrum, the lower is the standard mass spectrum in GC-MS database.

The peaks for t_R = 8.300 min is:

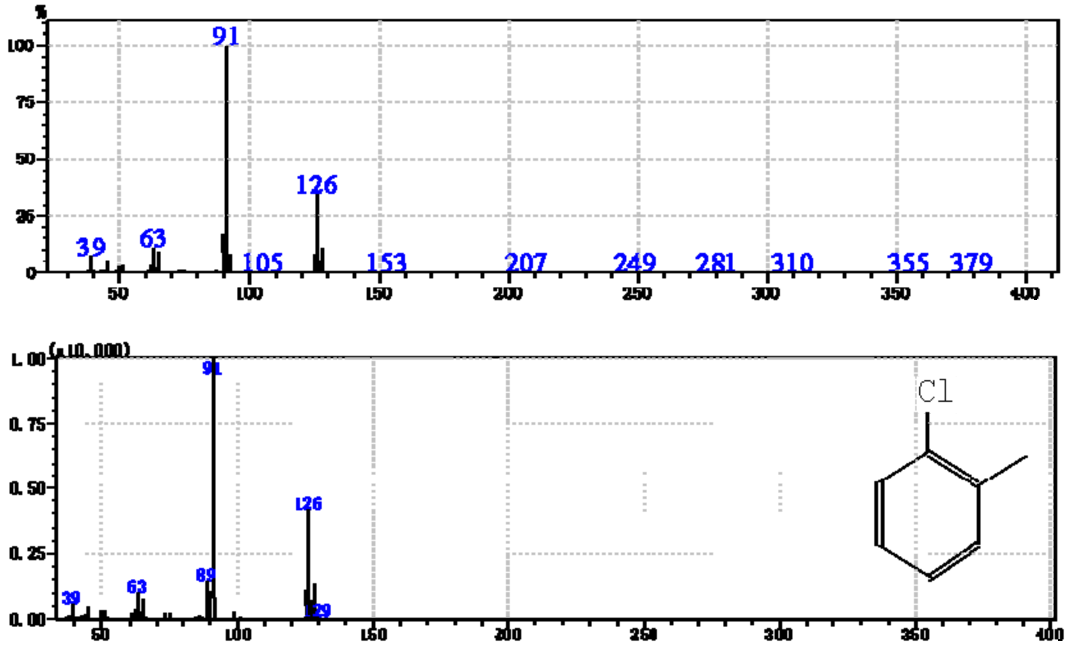

GC-MS: calcd. for C_7H_7Cl 126, found 126 (C_7H_7Cl). Table 2.

The peaks for t_R = 8.602 min is:

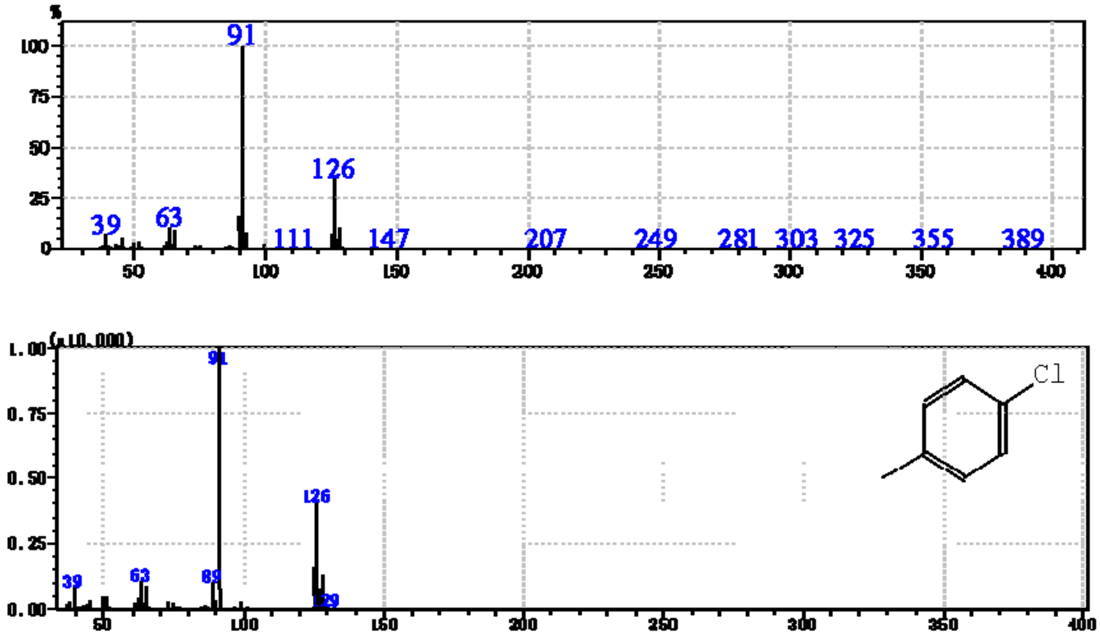

GC-MS: calcd. for C_7H_7Cl 126, found 126 (C_7H_7Cl). Table 2.

(2) Entry 5, Table 2

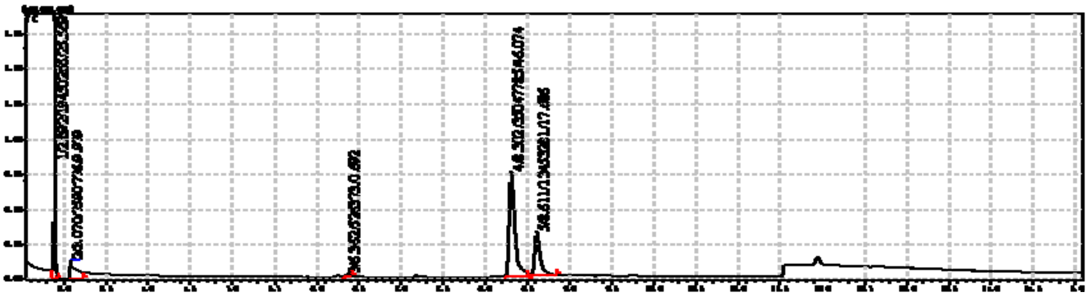

Figure S2. GC part of GC-MS for Entry 5, Table 2.

The peaks for t_R = 2.892 min and 3.070 are both unreacted toluene, the peak for t_R = 6.362 min is impurities from column, the peak for t_R = 8.302 min is 2-chlorotoluene, while the peak for t_R = 8.611 min is 4-chlorotoluene.

(3) Entry 8, Table 2

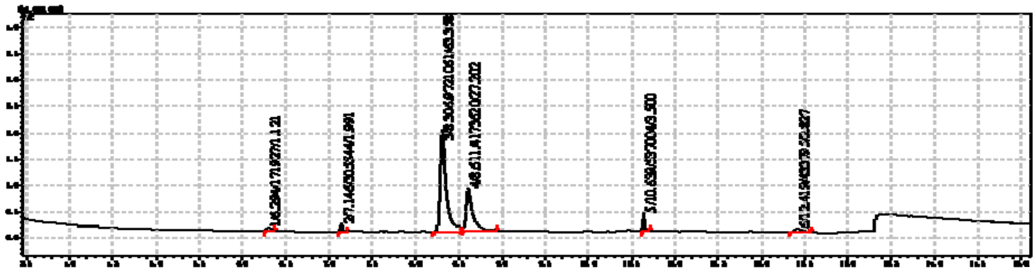

Figure S3. GC part of GC-MS for Entry 8, Table 2.

The peak for t_R = 8.306 min is 2-chlorotoluene, while the peak for t_R = 8.611 min is 4-chlorotoluene. Other very small peaks should be ascribed to impurities from column based on MS detection.

(4) Entry 9, Table 2

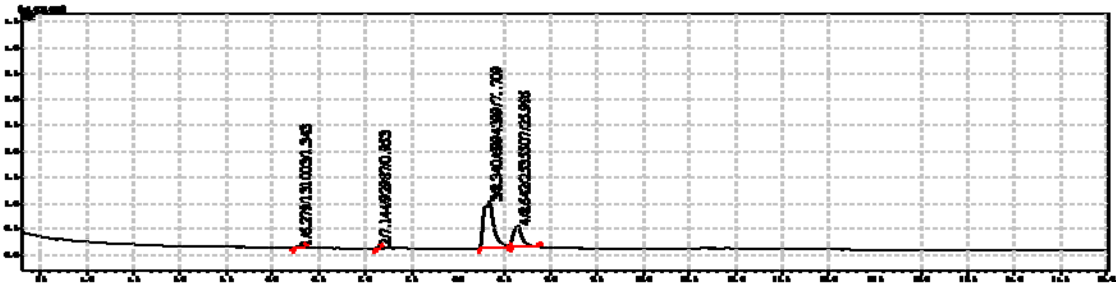

Figure S4. GC part of GC-MS for Entry 9, Table 2.

The peak for t_R = 8.340 min is 2-chlorotoluene, while the peak for t_R = 8.642 min is 4-chlorotoluene. Other very small peaks should be ascribed to impurities from column based on MS detection.

S2. GC-MS examples for Table 3

(1) Entry 3, Table 3

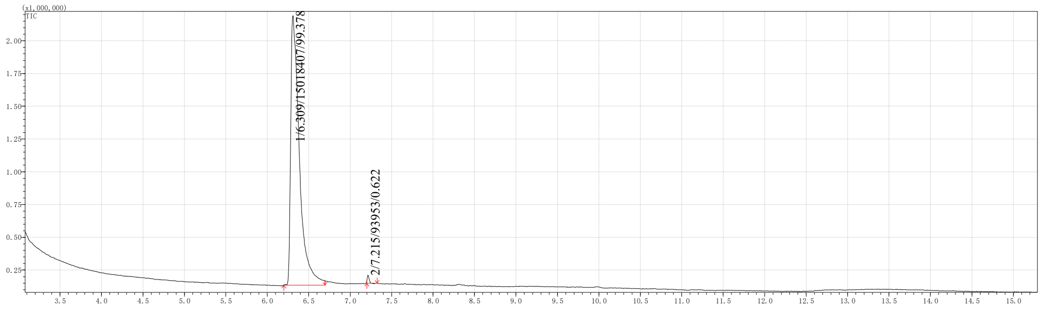

Figure S5. GC part of GC-MS for Entry 3, Table 3.

The peak for t_R = 6.309 min is:

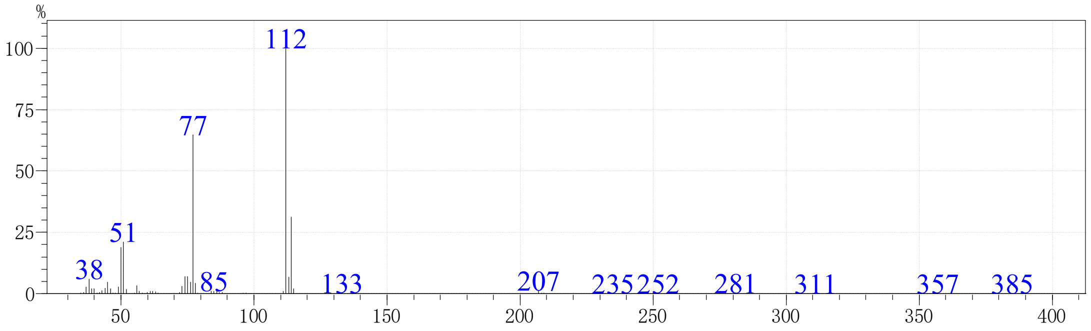

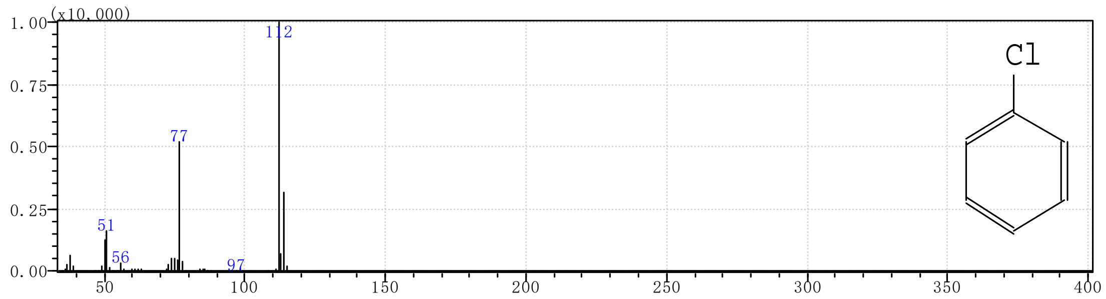

GC-MS: calcd. for C_6H_5Cl 112, found 112 (C_6H_5Cl). Table 3.

The peak for t_R = 7.219 min is impurities from column.

(2) Entry 5, Table 3

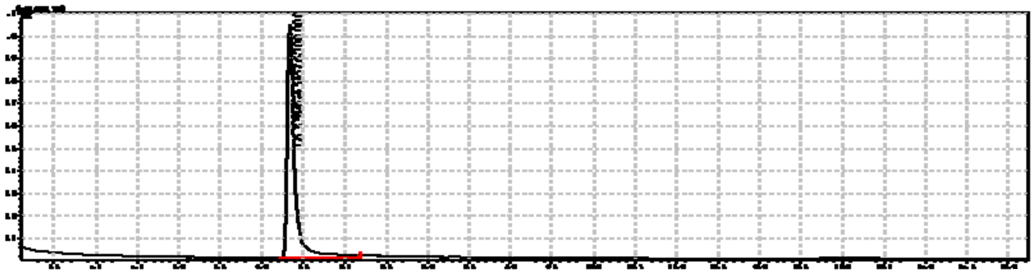

Figure S6. GC part of GC-MS for Entry 5, Table 3.

The peak for t_R = 6.338 min is:

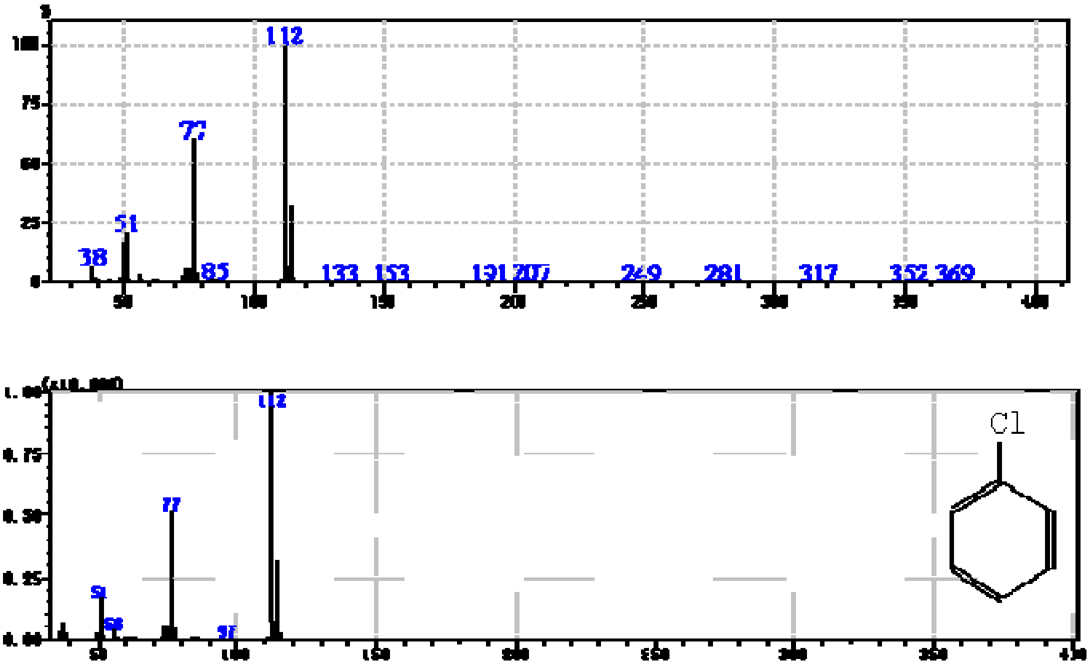

GC-MS: calcd. for C_6H_5Cl 112, found 112 (C_6H_5Cl). Table 3.

S3. GC-MS examples for Table 4

(1) Entry 1, Table 4

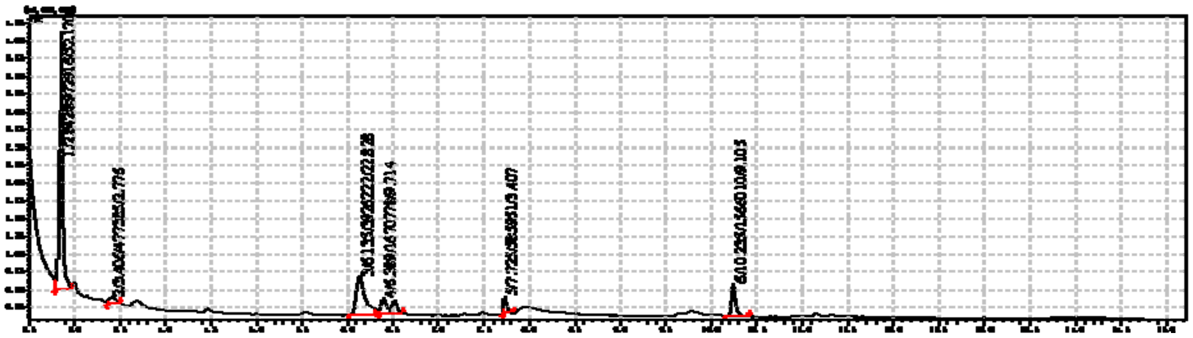

Figure S7. GC part of GC-MS for Entry 1, Table 4.

The peak for t_R = 2.842 min is:

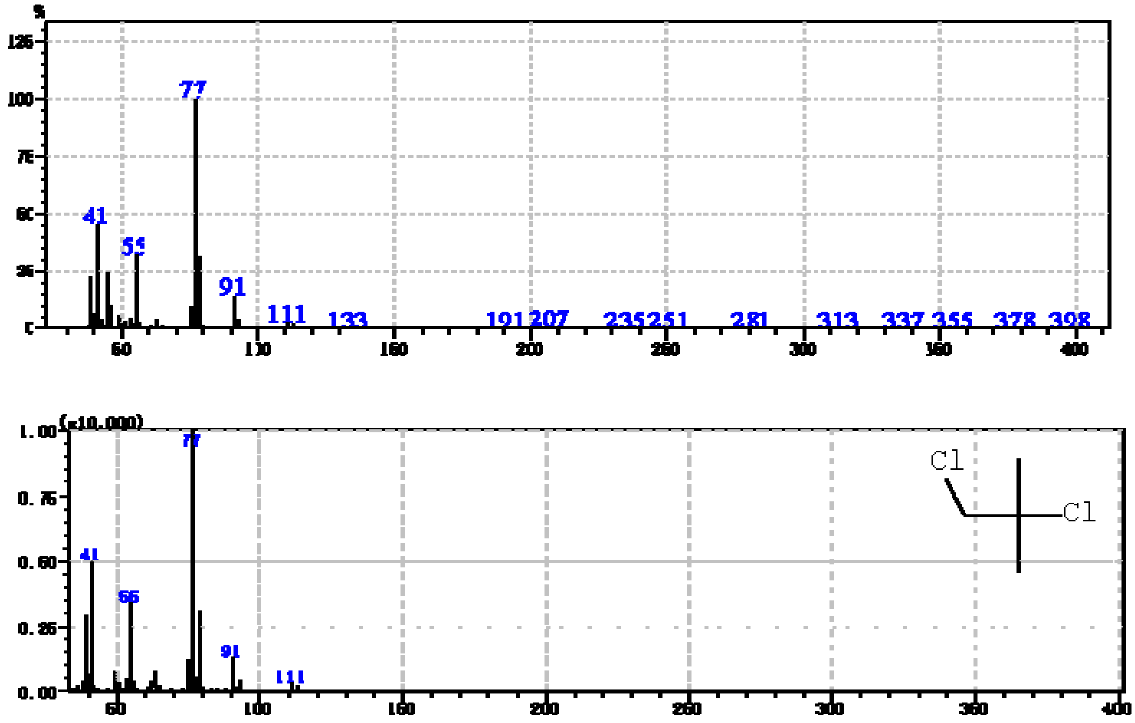

The peak for t_R = 6.135 min is:

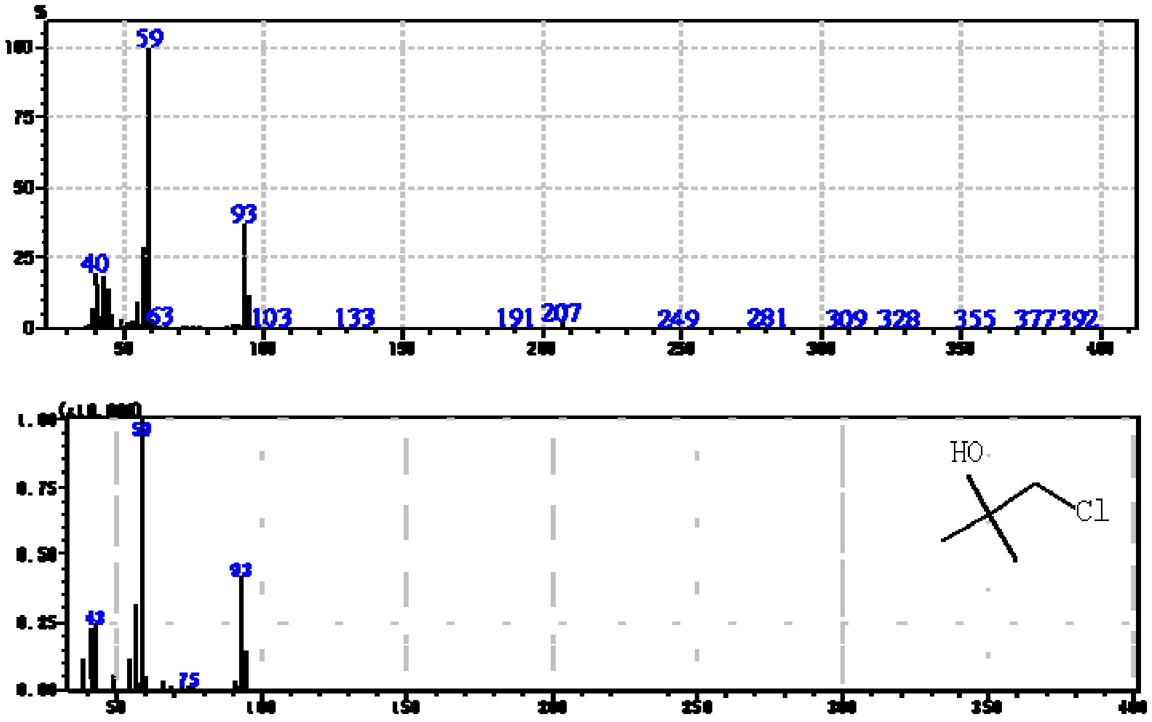

The peak for t_R = 10.235 min is:

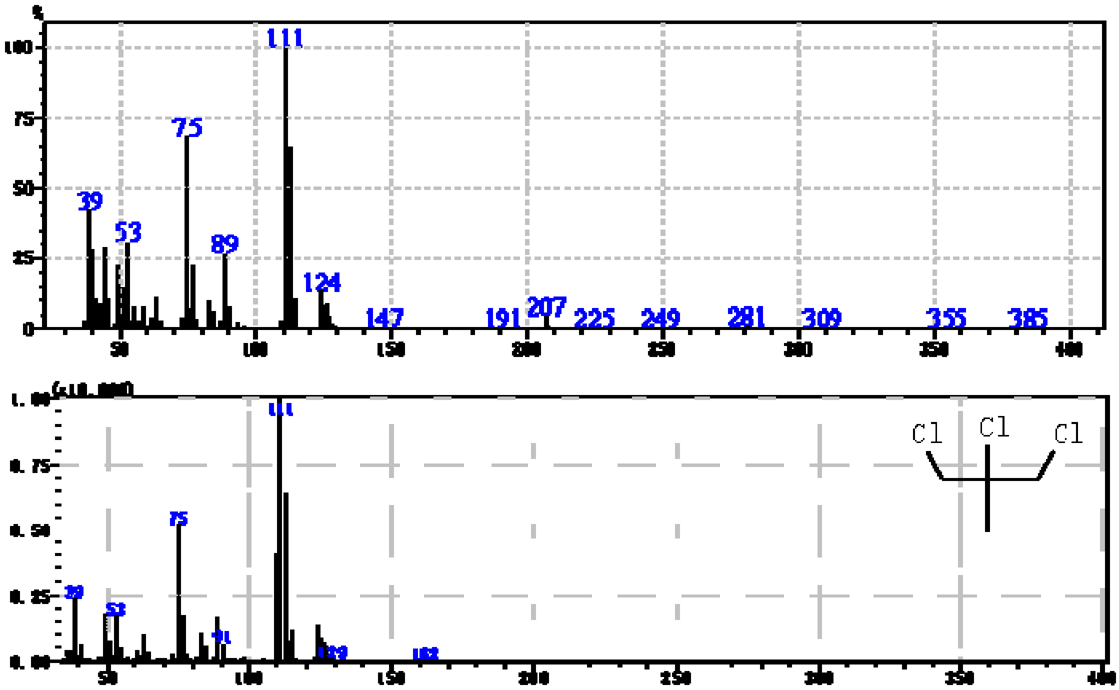

The other three peaks are impurities from column according to MS detection.

S4. GC-MS examples for Table 5

(1) Entry 1, Table 5

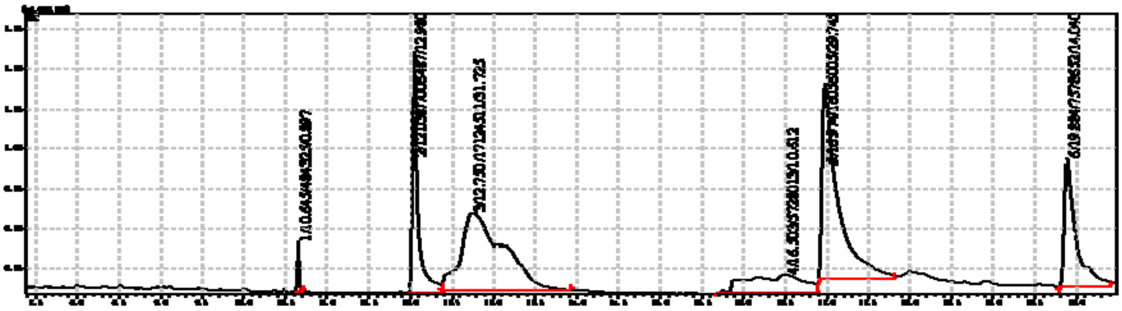

Figure S8. GC part of GC-MS for Entry 1, Table 5.

The peaks for t_R = 12.038 min and 12.750 are both:

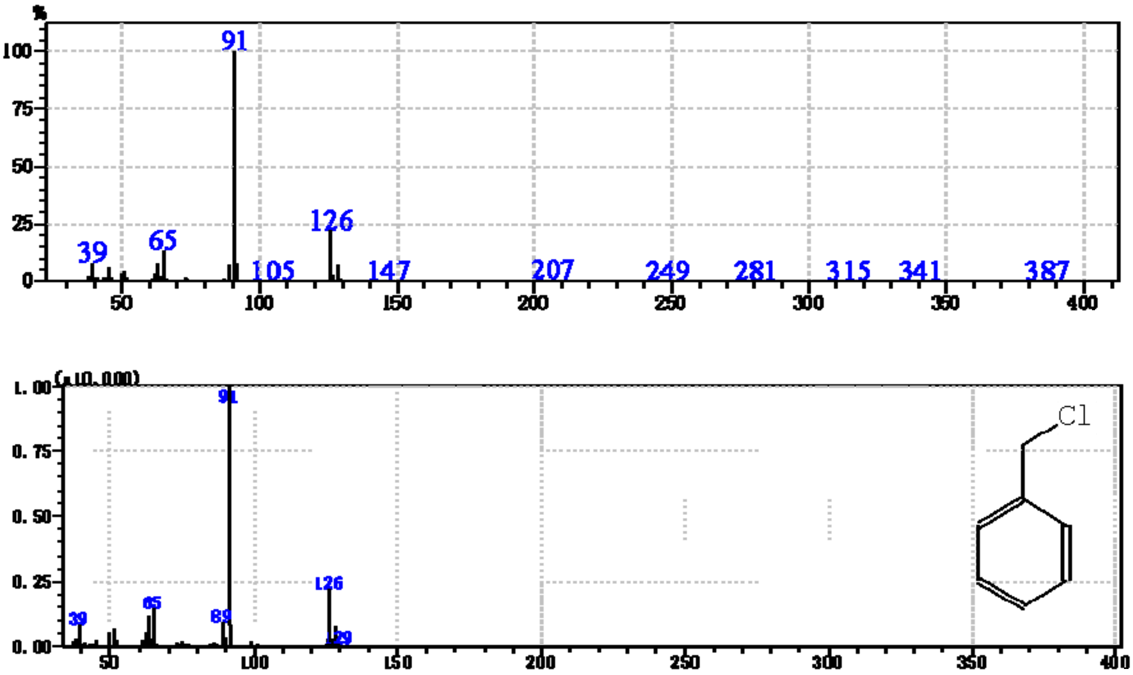

The peak for t_R = 16.503 min is:

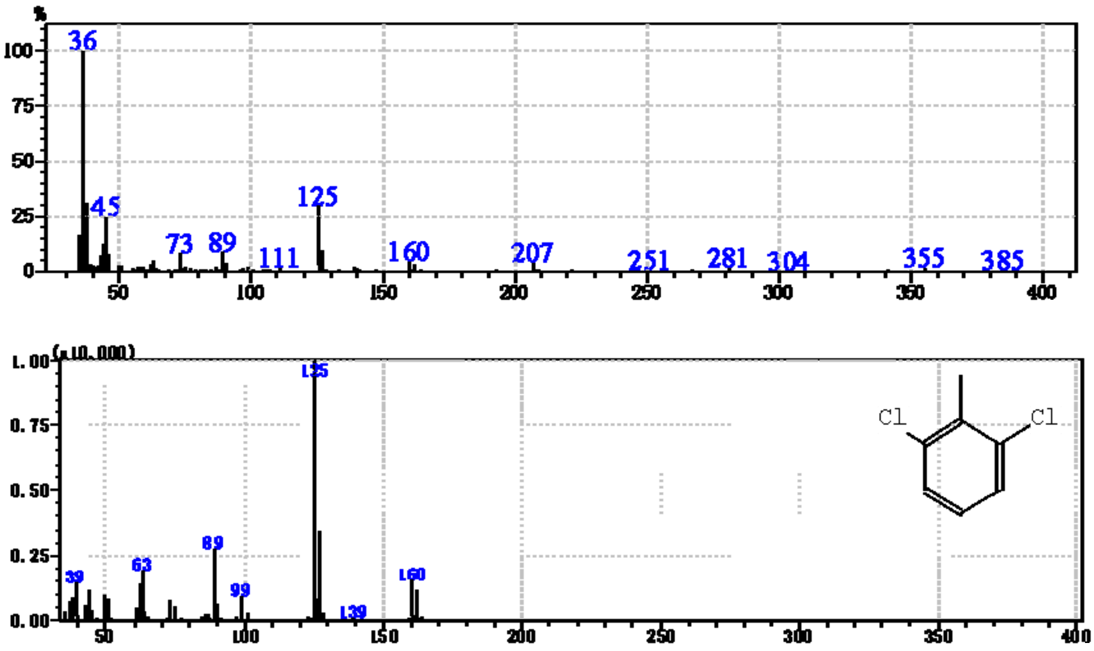

The peak for t_R = 16.974 min is:

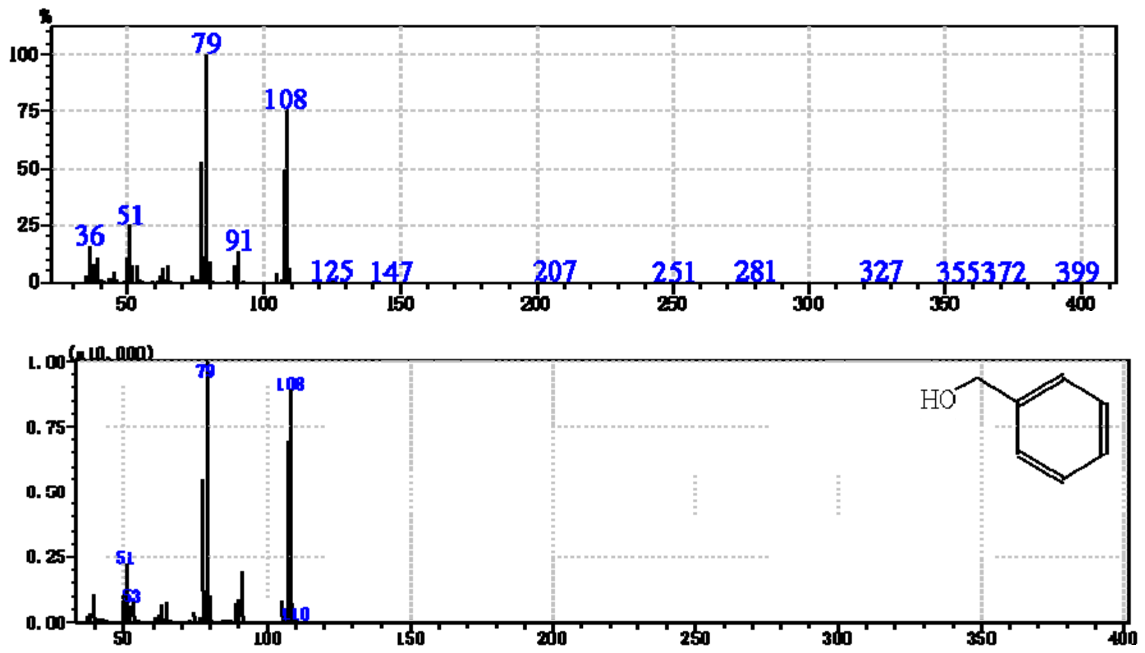

The peak for t_R = 19.884 min is:

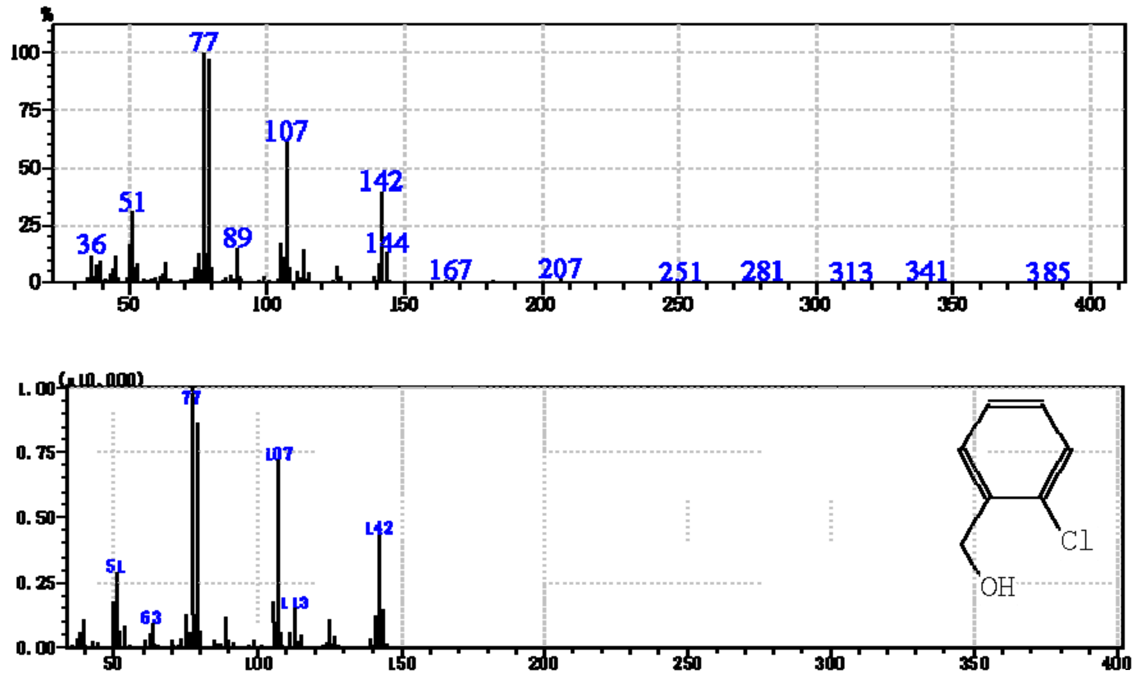

(2)Entry 2, Table 5

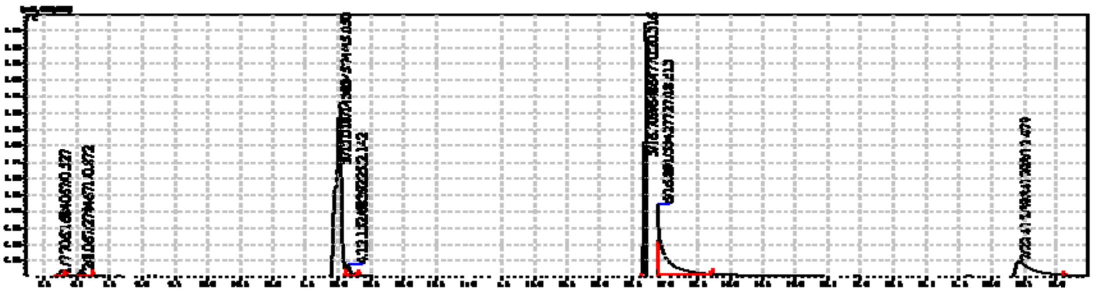

Figure S9. GC part of GC-MS for Entry 1, Table 5.

The peak for t_R = 12.018 min is:

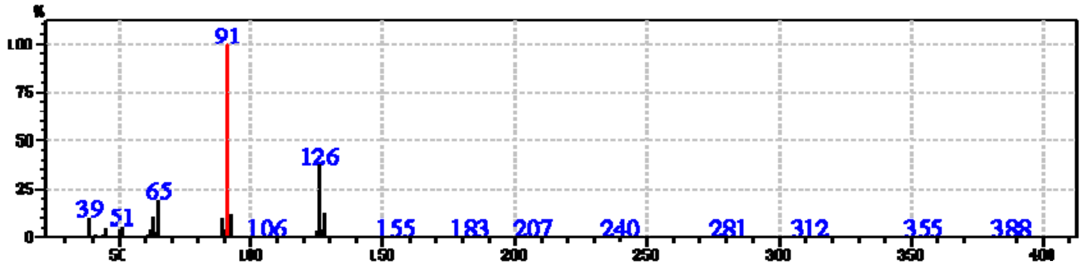

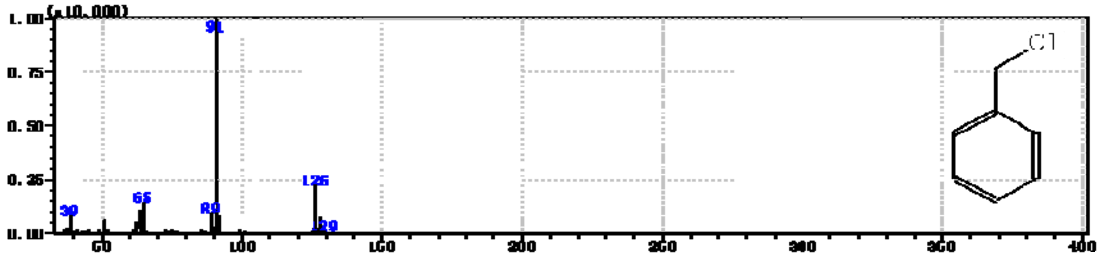

The peak for t_R = 12.152 min is:

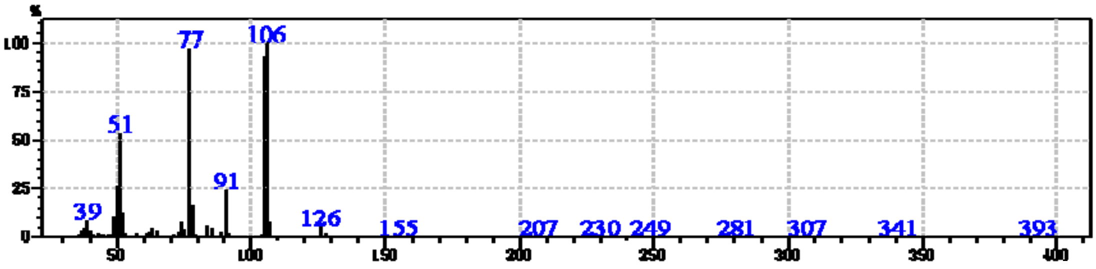

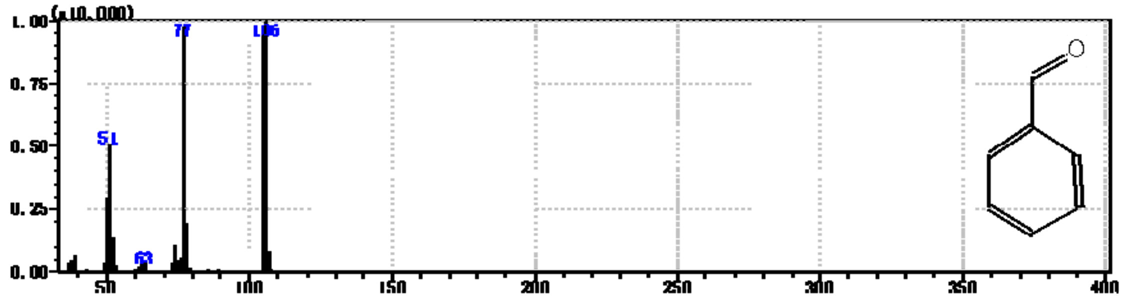

The peaks for t_R = 16.709 min and 16.891 are both:

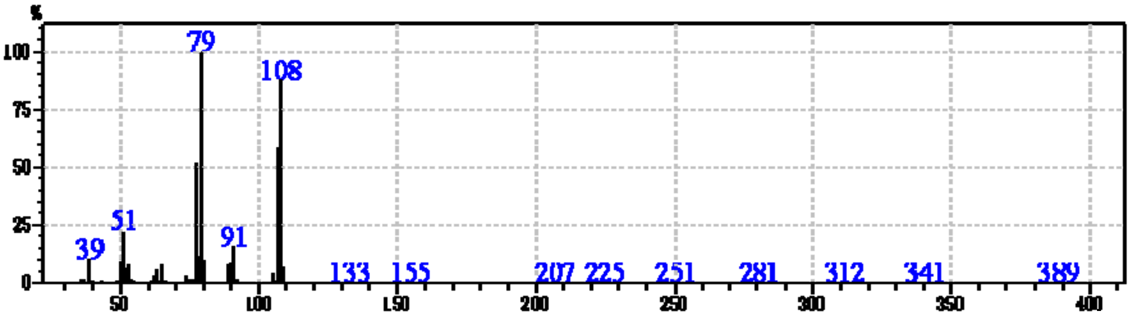

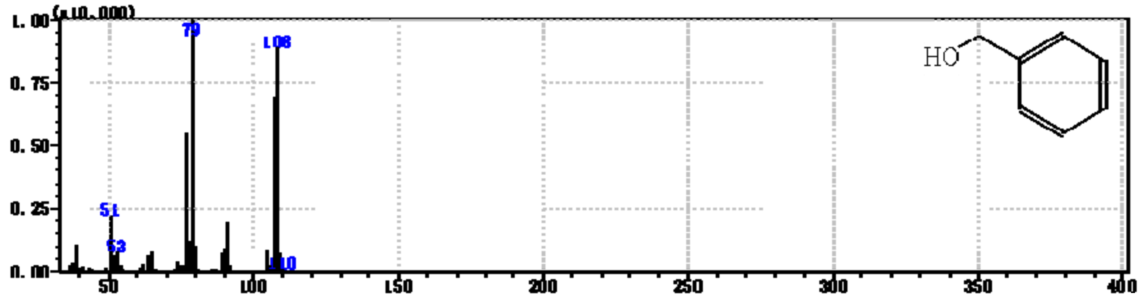

The peak for t_R = 22.415 min is:

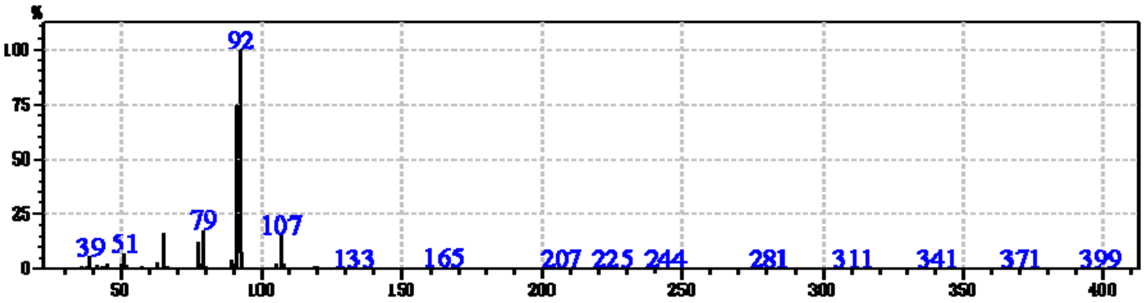

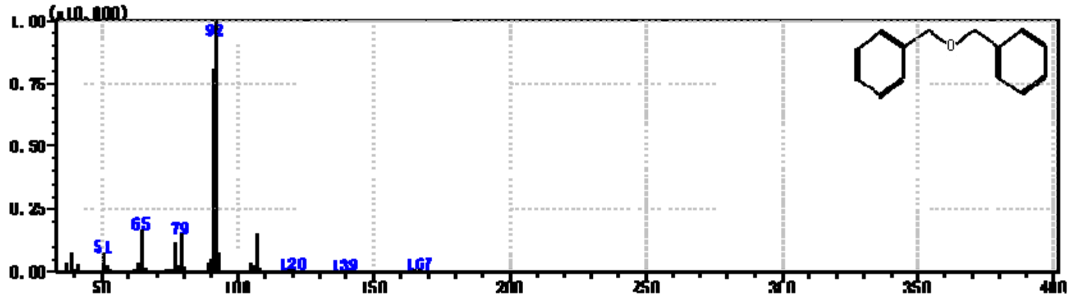

S5. GC-MS examples for Table 6

(1) Entry 1, Table 6

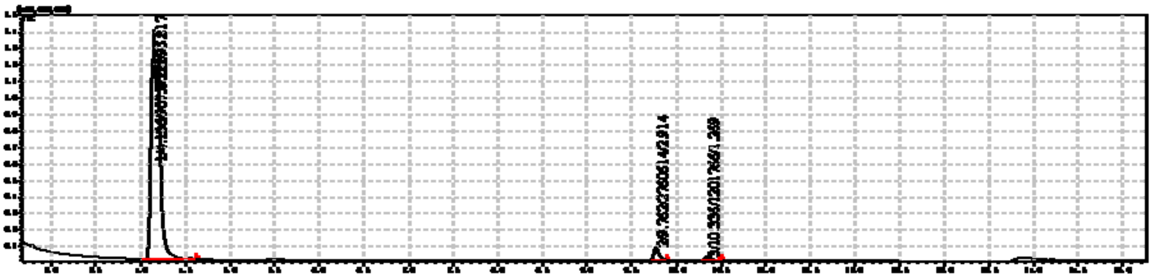

Figure S10. GC part of GC-MS for Entry 1, Table 6.

The peak for t_R = 4.106 min is:

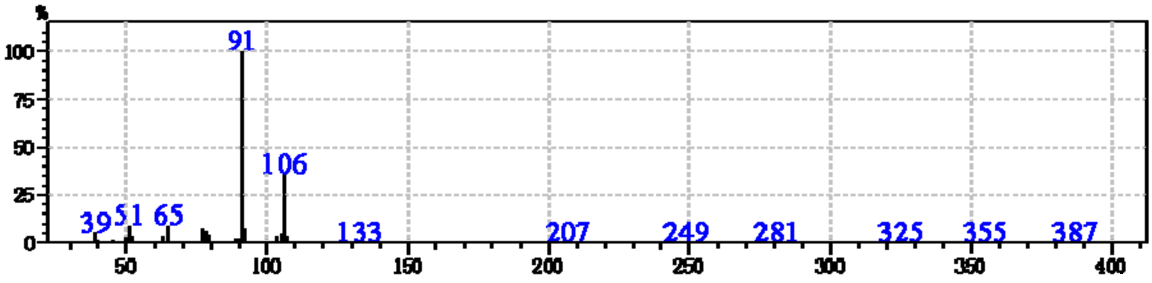

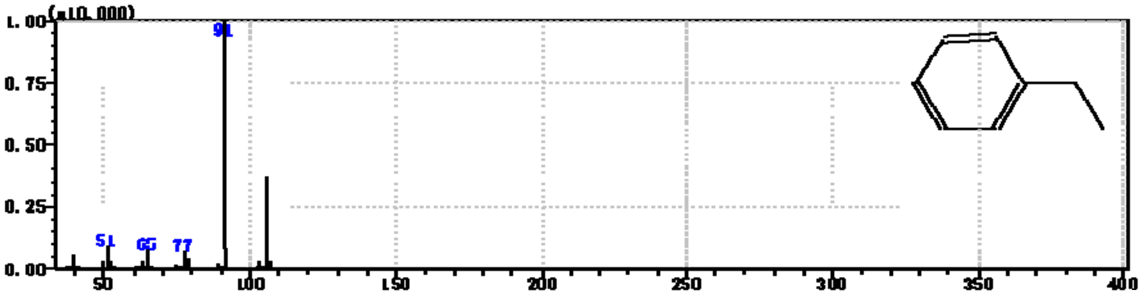

The peak for t_R = 9.762 min is:

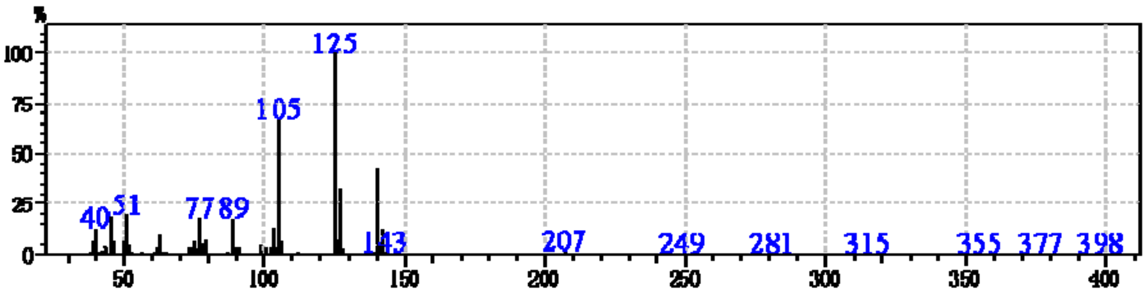

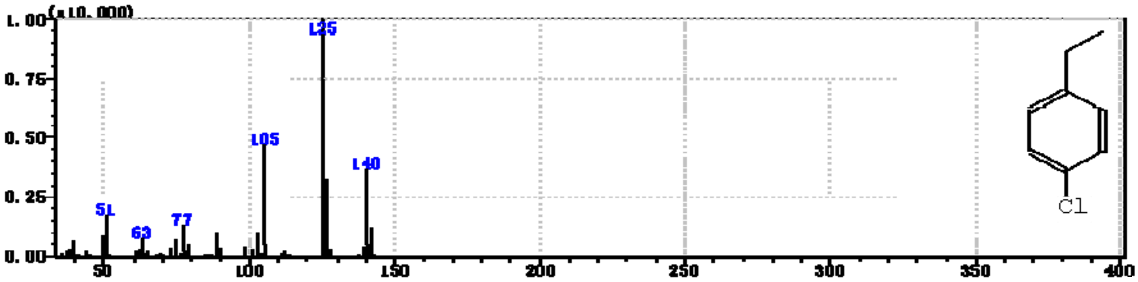

The peak for t_R = 10.336 min is:

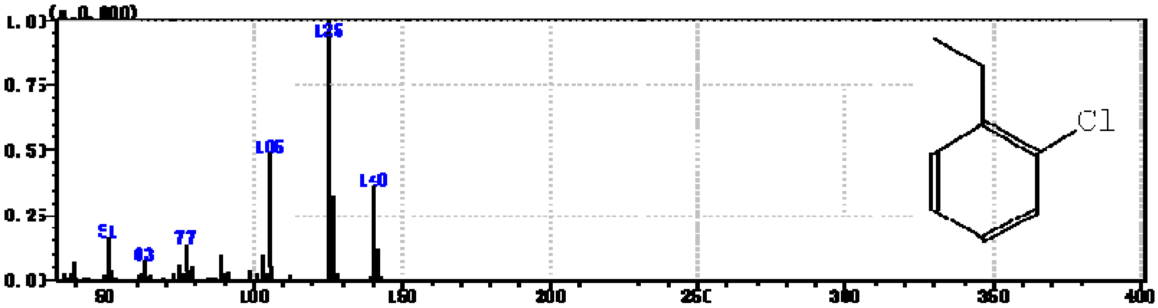

(2) Entry 3, Table 6

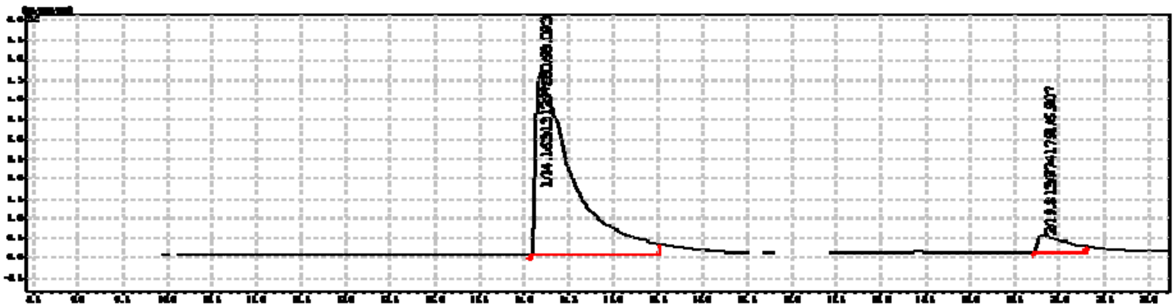

Figure S11. GC part of GC-MS for Entry 3, Table 6.

The peak for t_R = 14.163 min is:

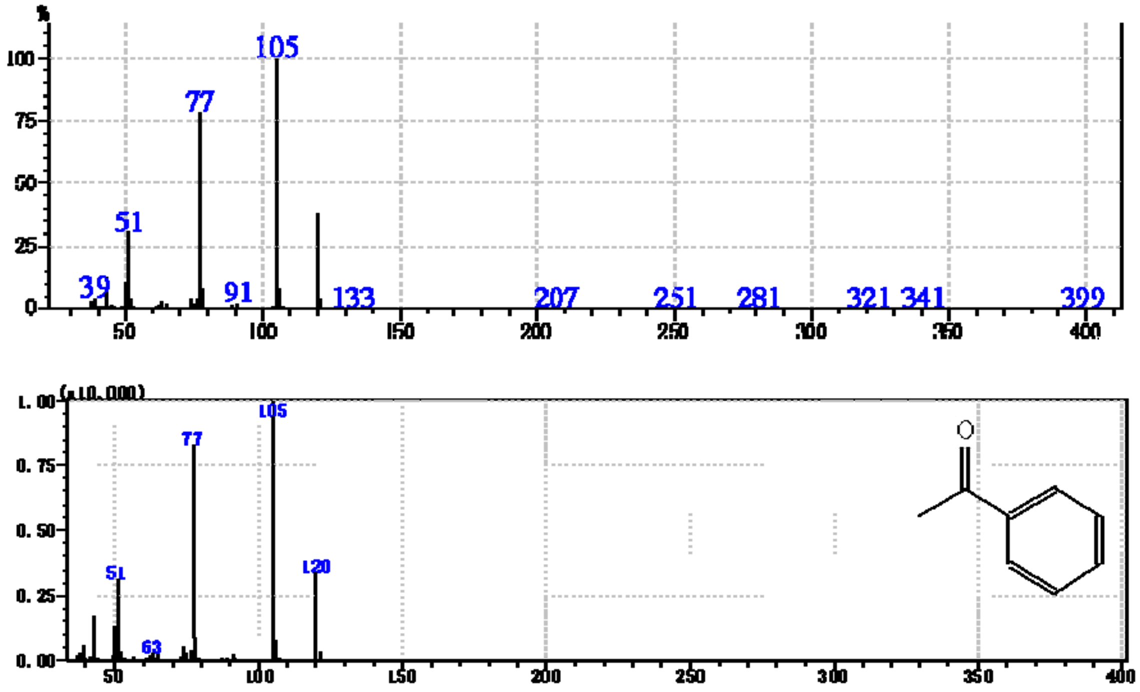

The peak for t_R = 19.813 min is:

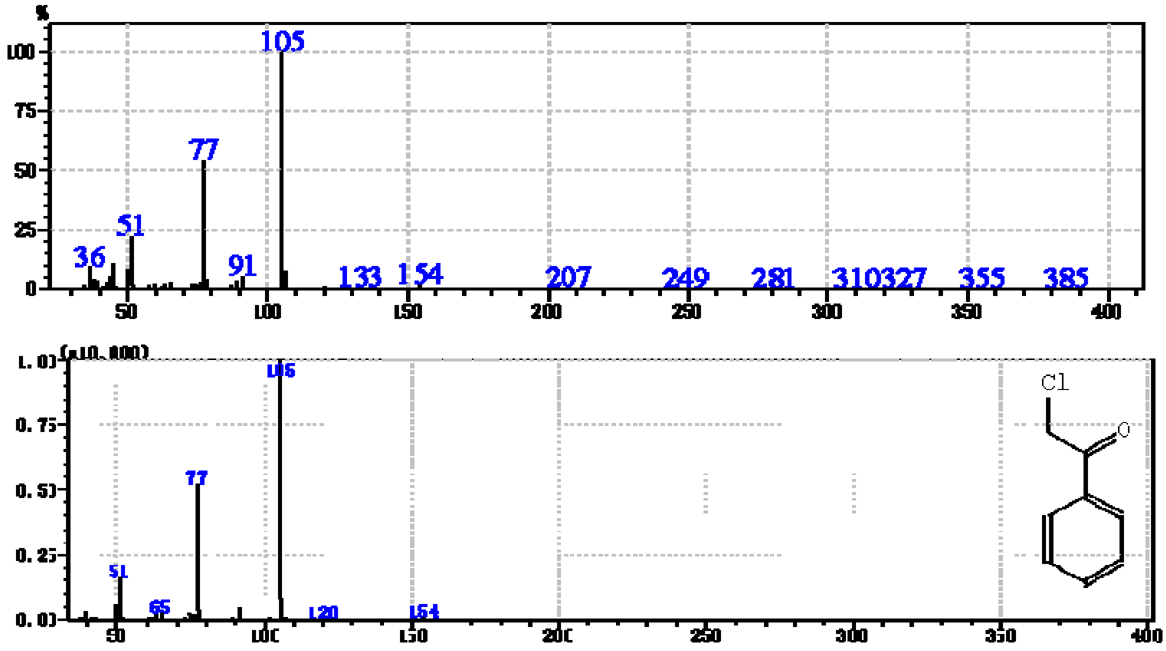

(3) Entry 4, Table 6

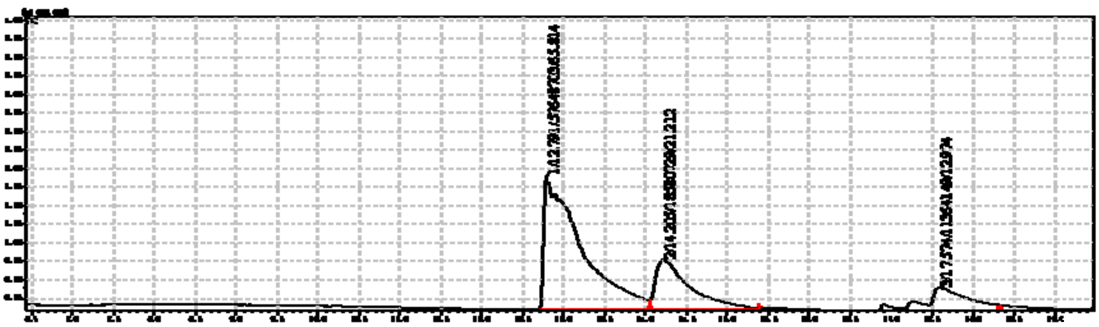

Figure S12. GC part of GC-MS for Entry 4, Table 6.

The peak for t_R = 12.791 min is:

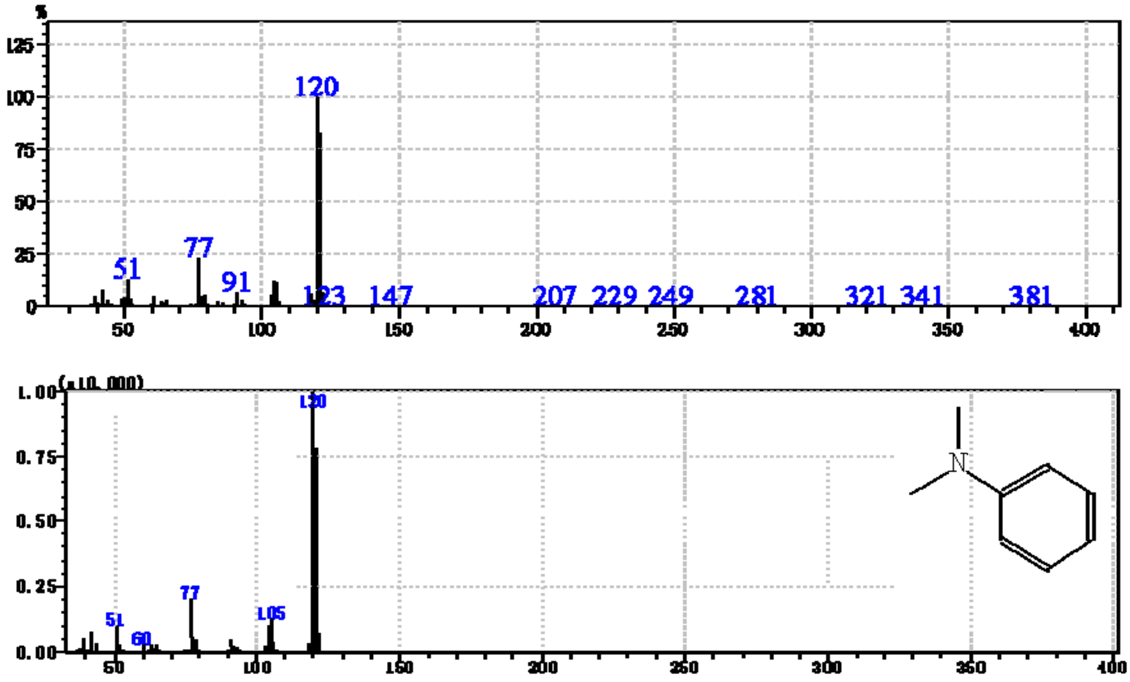

The peak for t_R = 14.205 min is:

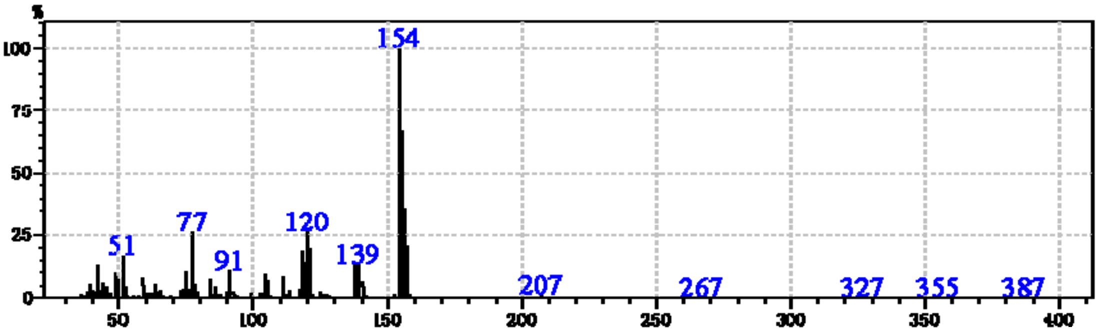

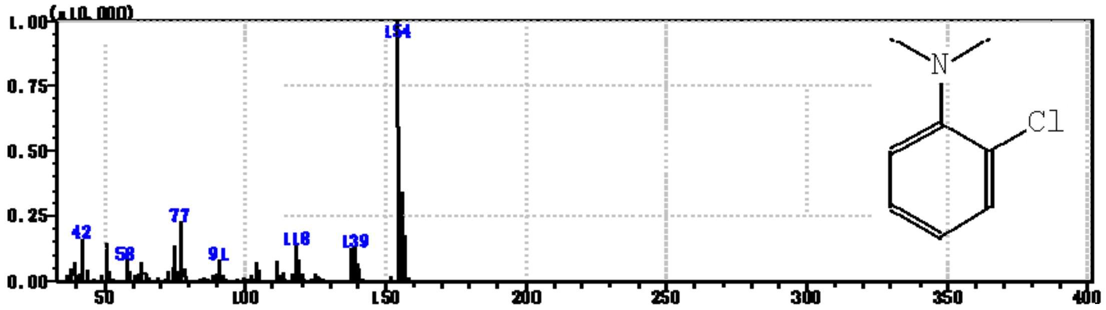

The peak for t_R = 17.574 min is:

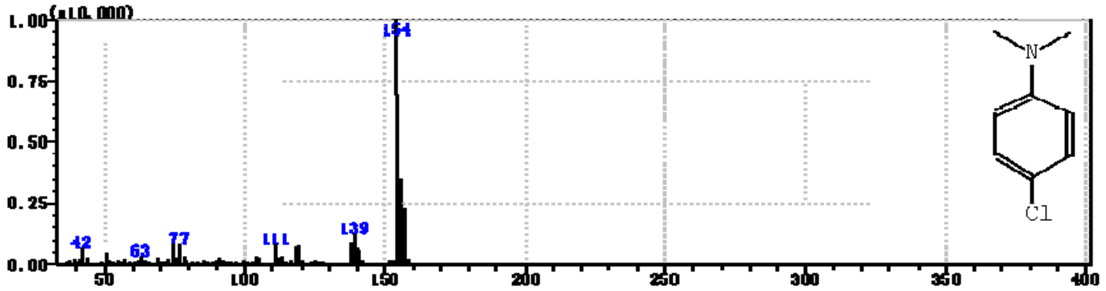

(4) Entry 5, Table 6

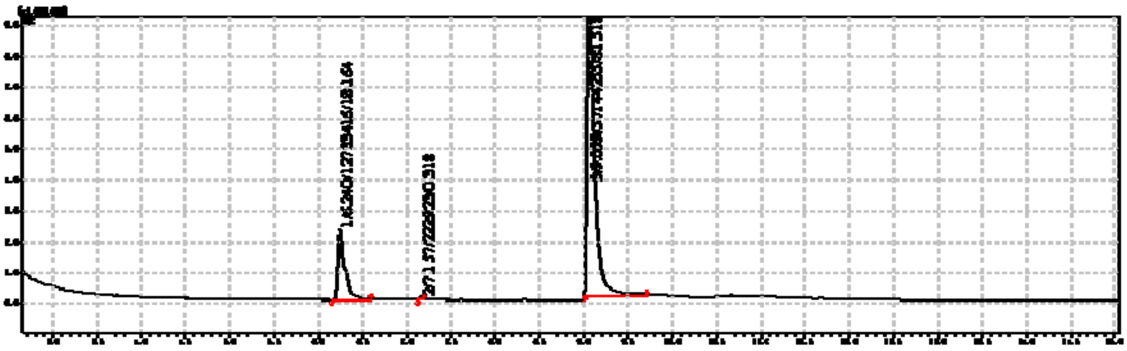

Figure S13. GC part of GC-MS for Entry 5, Table 6.

The peak for t_R = 6.240 min is:

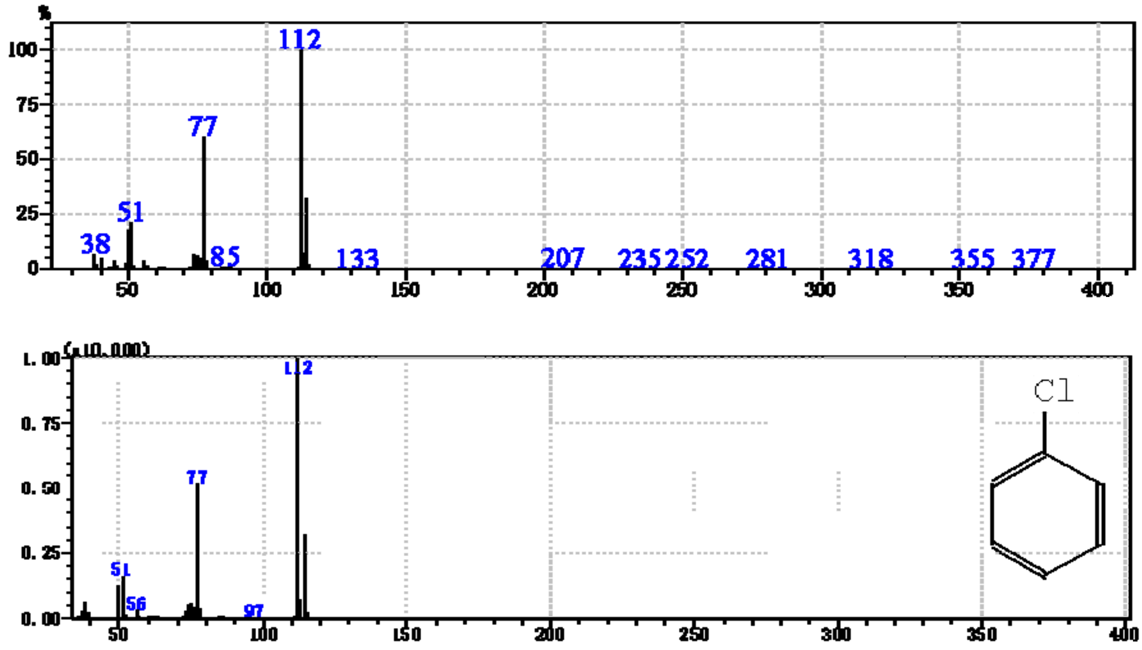

The peak for t_R = 9.059 min is:

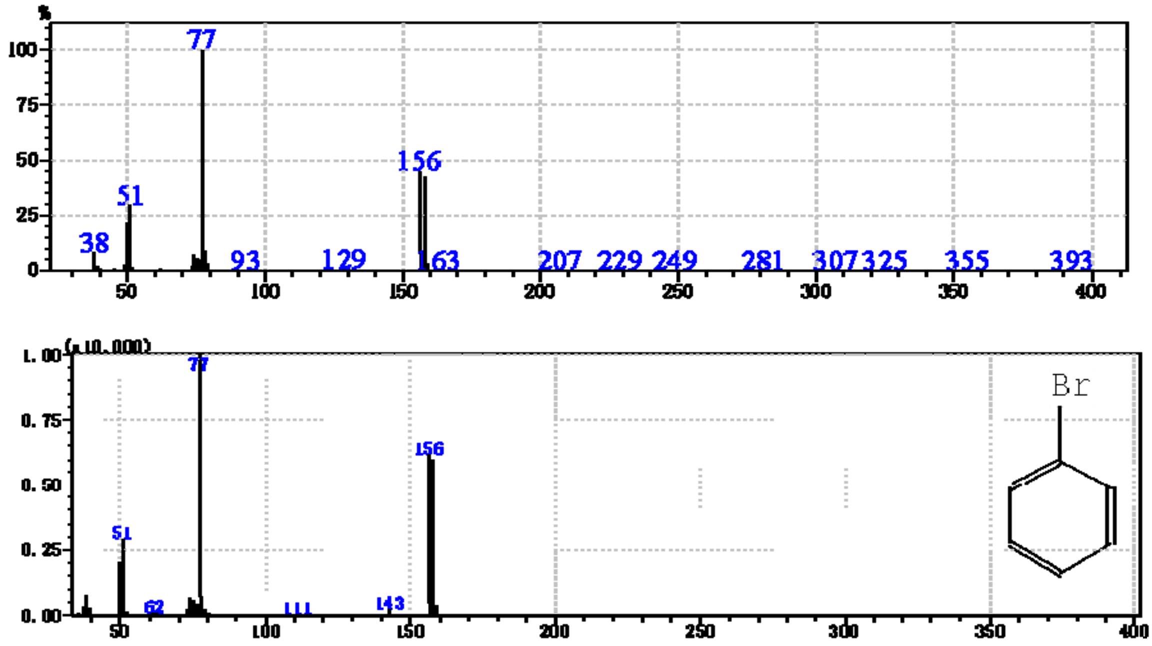

Supplement: Supplementary file 1 [file molecules-29-05451-s001.zip › molecules-3302158-supplementary.pdf]
